# Fluorinated Benzodipyrrole-Based Non-Fullerene Acceptors with Chlorinated End Groups Exhibiting Fluorine–Chlorine Interactions for Suppressed Charge Recombination in Organic Photovoltaics

**DOI:** 10.1021/acsami.5c23292

**Published:** 2026-02-05

**Authors:** Yan-Bo Wang, Yung-Jing Xue, Hong-Yi Chen, Chia-Lin Tsai, Jin Lee, Bing-Huang Jiang, Chih-Ping Chen, Fang-Chung Chen, Chain-Shu Hsu, Ta-Ya Chu, Jianping Lu, Su-Ying Chien, Yen-Ju Cheng

**Affiliations:** † Department of Applied Chemistry, 34914 National Yang Ming Chiao Tung University, 1001 University Road, Hsinchu 30010, Taiwan; ‡ Center for Emergent Functional Matter Science, National Yang Ming Chiao Tung University, 1001 University Road, Hsinchu 30010, Taiwan; § Department of Materials Engineering and Organic Electronics Research Center, Ming Chi University of Technology, New Taipei City 24301, Taiwan; ∥ Department of Photonics, National Yang Ming Chiao Tung University, 1001 University Road, Hsinchu 30010, Taiwan; ⊥ Quantum and Nanotechnologies Research Centre, 6356 National Research Council Canada, 1200 Montreal Road, Ottawa, Ontario, Canada; # Instrumentation Center, 33561 National Taiwan University, No.1, Sec. 4, Roosevelt Road, Taipei 10617, Taiwan

**Keywords:** benzodipyrrole core, chlorination, non-fullerene acceptor, organic solar cell, ternary strategy

## Abstract

Two terminal-chlorinated *ortho*-benzodipyrrole (*o*-BDP)-based non-fullerene acceptors (NFAs), CFB-Cl and CMB-Cl, were designed and synthesized by incorporating fluorine or methyl substituents on the *o*-BDP core, respectively. Compared to their terminal-fluorinated counterparts, both NFAs exhibit red-shifted absorption, higher melting points, and stronger intermolecular interactions, attributed to the introduction of chlorinated end groups. Single-crystal X-ray analysis of CFB-Cl revealed a compact three-dimensional kaleidoscopic packing network stabilized by unique F···Cl halogen interactions between the fluorinated *o*-BDP core and the chlorinated end group, leading to a short π–π stacking distance of 3.38 Å and enhanced charge transport. Consequently, PM6:CFB-Cl devices achieved a PCE of 16.62% with a fill factor (FF) of 75.54%, outperforming PM6:CMB-Cl (PCE = 16.13%). To further improve device performance, a ternary blend strategy was employed by introducing the fluorinated CMB into PM6:CFB-Cl blends to extend the absorption range and improve the morphology. The resulting PM6:CFB-Cl:CMB inverted device exhibited excellent miscibility (χ = 0.02 K), balanced carrier transport (μ_e_/μ_h_ = 1.38), suppressed recombination, and a highest PCE of 17.26% with *J*_sc_ = 26.02 mA cm^–2^ and *V*_oc_ = 0.892 V. This work highlights the importance of halogen engineering in regulating molecular packing and charge dynamics, providing insights into the structure–morphology–performance relationship of o-BDP-based NFAs for next-generation organic photovoltaics.

## Graphical abstract

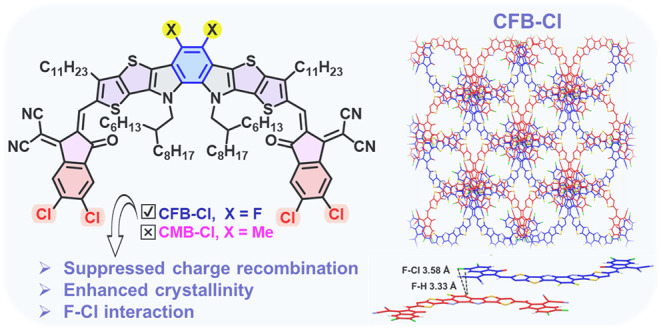

## 1 Introduction

Y6, a landmark non-fullerene acceptor (NFA) featuring an A-D_N_A′_N_D-A architecture and a C-shaped conformation, has revolutionized the field of organic photovoltaics (OPVs), achieving a remarkable power conversion efficiency (PCE) of 15.7% in 2019.^1^ Structurally, Y6 consists of a central *ortho*-benzodipyrrole (*o*-BDP) core vertically fused with an electron-deficient thiadiazole unit (A′) and laterally fused with two β-alkylated thieno­[3,2-*b*]­thiophene (TT) units in which the nitrogen atoms (N) serve as electron-rich bridges. The 2-(5,6-difluoro-3-oxo-2,3-dihydro-1*H*-indene-1-ylidene)­malononitrile (FIC) group is widely used as the terminal electron-accepting unit (A). The success of Y6 has inspired extensive research on the molecular engineering of the A-D_N_A′_N_D-A framework. Efforts have focused on side-chain modifications on the D units or N bridges,^2–7^ chalcogen substitution on the donor cores,^8,9^ variations of the central A′ heterocyclic units,^10–18^ and end-group engineering.^19–23^ These collective advances have propelled the PCEs of Y6 derivatives beyond 20%.^24–30^ Among these strategies, end-group engineering has been considered as the most straightforward and synthetically efficient approach.^20,31^ For instance, replacing the FIC unit with 2-(5,6-dichloro-3-oxo-2,3-dihydro-1*H*-indene-1-ylidene)­malononitrile (Cl-IC), possessing a stronger dipole moment due to the C–Cl bond, generally leads to red-shifted absorption and enhanced crystallinity compared to the fluorinated counterparts.^31–34^

Recently, we developed a new structurally simplified A-D_N_B_N_D-A type NFA, named CB16, which employs an unsubstituted *o*-BDP core derived from Y6 by removing the thiadiazole (A′) moiety.^35^ The “C” refers to the C-shaped geometry, and “B” signifies the central benzene ring. Despite the absence of the A′ unit, CB16 maintains a three-dimensional (3D) packing network reminiscent of that of Y6, resulting in efficiencies comparable to those of state-of-the-art Y6-based materials. The pivotal role of the C-shaped skeleton paves the way for the future development of *o*-BDP-based NFAs.^36–42^ The unsubstituted *o*-BDP core introduces two additional substitution sites on the central benzene ring, offering a versatile platform for further functionalization. Incorporating fluorine atoms or methyl groups on the *o*-BDP ring produced CFB and CMB, respectively, which yielded OPV devices with suppressed charge recombination and reduced energy loss.^40^ Notably, single-crystal analysis of CFB revealed intermolecular F···F interactions between the fluorinated *o*-BDP core and the FIC terminal units, highlighting the critical role of halogen interactions in molecular packing.^40^

Halogen engineering using chlorine substitution has also gained increasing attention as an effective strategy to fine-tune energy levels, intramolecular charge transfer, and intermolecular interactions.^32–34^ Compared to fluorine, chlorine exhibits several intrinsic differences, including a decrease in electronegativity from 4.0 for fluorine to 3.16 for chlorine and an increase in atomic size from 0.071 nm for fluorine to 0.102 nm for chlorine. The larger and more diffuse outer electron cloud of chlorine makes it highly polarizable, potentially enhancing intermolecular interactions through efficient π/p-orbital overlap. This improved orbital interaction is expected to enhance crystallinity and charge-transport properties in conjugated materials.^43–45^ More importantly, the higher polarizability of the chlorine atom can lead to an increased dielectric constant, which in turn reduces the exciton binding energy, suppresses charge recombination, and enhances charge extraction efficiency.^41,43^

In this study, we further extended this molecular design by condensing the difluoro- and dimethyl-substituted *o*-BDP backbones with the chlorinated terminal group (Cl-IC), affording two new NFAs: CFB-Cl and CMB-Cl (Figure 1). Compared with their FIC-terminated analogues (CFB and CMB), both CFB-Cl and CMB-Cl exhibit markedly red-shifted absorption, higher melting temperatures, and stronger intermolecular interactions. Single-crystal analysis revealed that CFB-Cl forms a compact 3D network stabilized by unique F···Cl interactions. Consequently, PM6:CFB-Cl devices achieved a high fill factor (FF) of 75.54% and a high PCE of 16.62%, surpassing PM6:CMB-Cl devices (PCE = 16.13% and FF = 68.04%).

**Figure 1:**
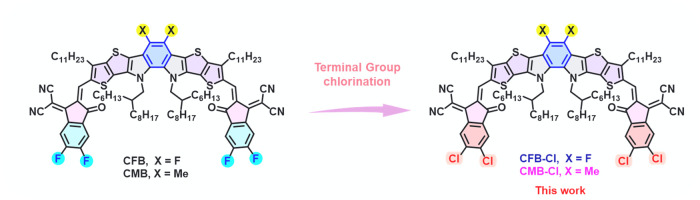
Chemical structures of CFB, CMB, CFB-Cl, and CMB-Cl.

Besides molecular engineering, the acceptor alloy strategy, which involves blending two distinct non-fullerene acceptors (NFAs) within the active layer, has emerged as an effective approach to enhance the efficiency of OPV devices by combining the advantages of each acceptor component.^46–54^ In the ternary PM6:CFB-Cl:CMB blend, the formation of a CFB-Cl:CMB acceptor alloy leverages the complementary properties of the individual acceptors, including optimized energy level alignment, broadened absorption, and favorable molecular packing. These synergistic effects collectively enable enhanced light harvesting, more balanced charge transport, reduced energy losses, and improved morphological stability, resulting in a significantly increased PCE of 17.26% with a *J*_sc_ exceeding 26 mA cm^–2^.

## 2 Results and Discussion

### 2.1 Molecular Synthesis and Characterization

The synthetic routes of CFB-Cl and CMB-Cl are depicted in Scheme 1. Starting materials 1a and 1b were synthesized according to the literature.^40^ Cyclization of compounds 1a and 1b using Pd-catalyzed Buchwald–Hartwig amination with 2-hexyldecyl amine resulted in the formation of compounds 2a and 2b, which were further formylated by Vilsmeier reagent to afford compounds 3a and 3b, respectively. Knoevenagel condensation of compounds 3a and 3b with Cl-IC acceptor yielded the final products, CFB-Cl and CMB-Cl, respectively. The characterization data and the detailed synthetic procedures are provided in Supporting Information (Figures S9–S13).

**Scheme 1:**
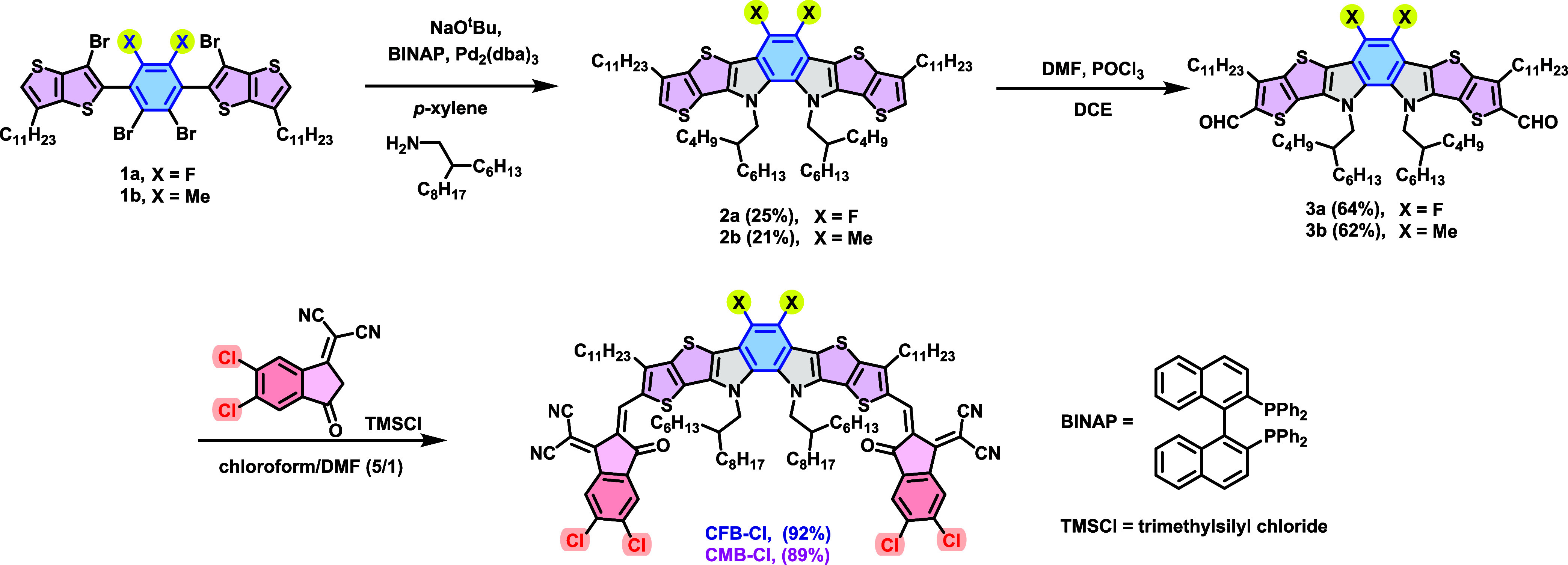
Synthetic Routes of CFB-Cl and CMB-Cl

### 2.2 Thermal Properties

Thermogravimetric analysis (TGA) and differential scanning calorimetry (DSC) were performed to obtain the thermal properties of the acceptors (Figure S1 and Figure 2a). Both CFB-Cl and CMB-Cl display a decomposition temperature (*T*_d_) over 320 °C, resembling that of their terminal-fluorinated analogues. In addition, the chlorination on the terminal group also leads to a significantly higher melting temperature (*T*_m_) for both CFB-Cl (269 °C) and CMB-Cl (251 °C) compared to CFB (224 °C) and CMB (222 °C), indicating the enhanced intermolecular interaction resulting from the chlorinated IC unit.

**Figure 2:**
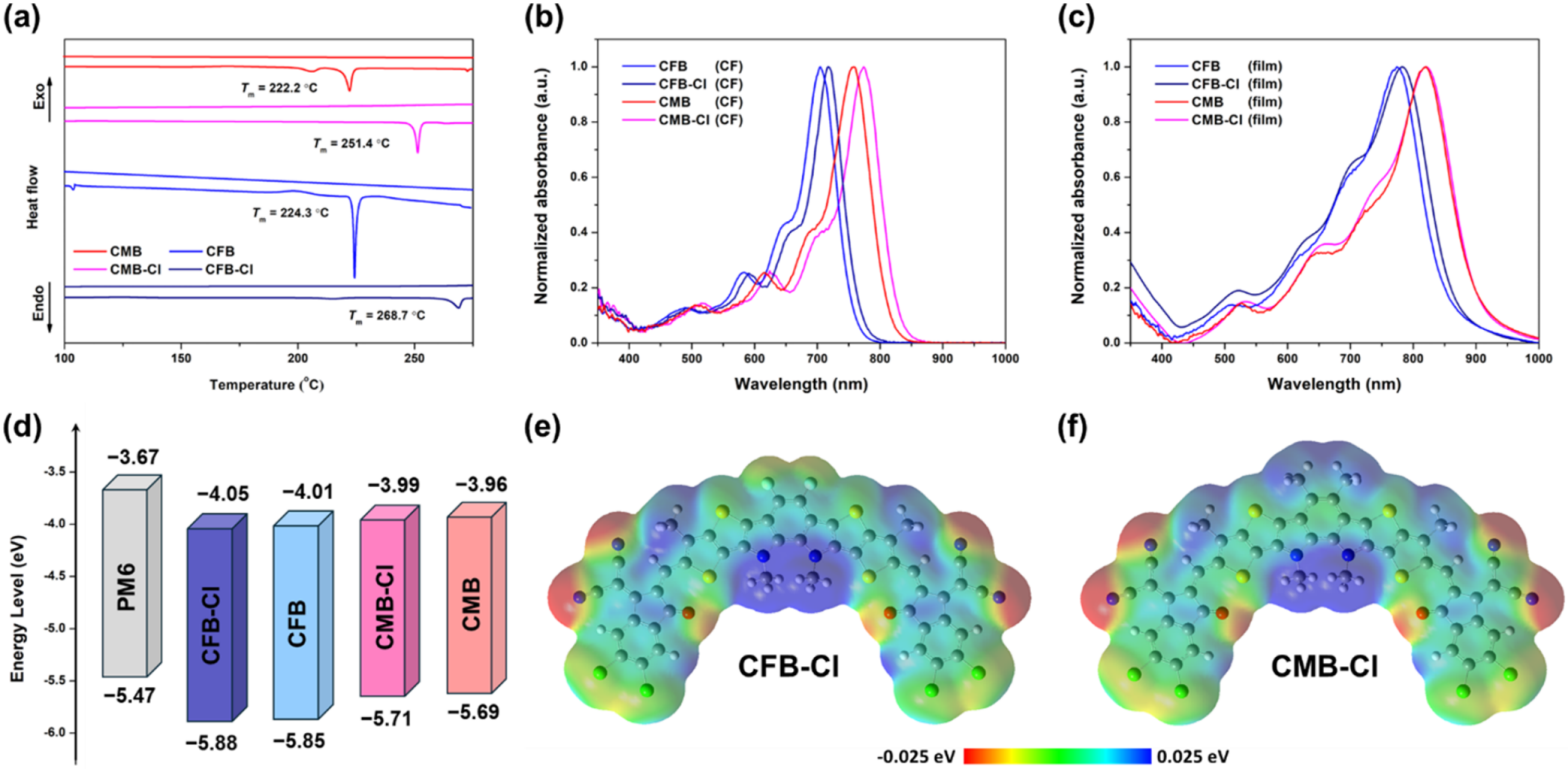
(a) DSC measurement results of CFB-Cl, CFB, CMB-Cl, and CMB with a ramping rate of 10 °C min^–1^. UV–vis absorption spectrum of the NFAs measured in (b) solution and (c) thin film state. (d) Energy level diagrams of PM6 and the NFAs. Electrostatic potential diagrams of (e) CFB-Cl and (f) CMB-Cl.

### 2.3 Optical and Electrochemical Measurements

Absorption spectra in both chloroform and film states were measured for CFB, CFB-Cl, CMB, and CMB-Cl and are shown in Figure 2b,c, with their intrinsic properties summarized in Table 1. In the chloroform solution, the maximum absorption peaks (λ_max_) of CFB-Cl and CMB-Cl are observed at 717 and 774 nm, respectively. The blue-shifted absorption of CFB-Cl indicates that incorporating the electron-withdrawing fluorine atoms on the *o*-BDP unit weakens the electron-donating capability of the D_N_B_N_D moiety, thereby reducing the intramolecular charge transfer (ICT). Compared with fluorinated end-group counterparts, CFB-Cl and CMB-Cl show more red-shifted absorption features. These results indicate that a larger dipole moment of the C–Cl bond on the chlorinated IC unit facilitates a more efficient ICT effect, leading to red-shifted absorption. In the film state, both CFB-Cl and CMB-Cl exhibit much red-shifted absorption peaks with λ_max_ located at 782 and 823 nm, implying strong intermolecular interactions in the film state. It is noted that CMB and CMB-Cl exhibit similar λ_max_ values in the thin film state, presumably due to the steric hindrance of methyl groups. The HOMO/LUMO energy levels of CFB-Cl and CMB-Cl were estimated through cyclic voltammetry measurements (Figure S2), and the results were demonstrated as an energy level diagram in Figure 2d. The HOMO/LUMO energy levels were −5.85/–4.01, −5.88/–4.05, −5.69/–3.96, and −5.71/–3.99 eV for CFB, CFB-Cl, CMB, and CMB-Cl, respectively. Both CFB-Cl and CMB-Cl display downshifted HOMO and LUMO energy levels compared with their terminal-fluorinated analogues. Chlorination of CFB-Cl and CMB-Cl has a more pronounced effect on the LUMO energy level than on the HOMO energy level, leading to the narrower bandgaps, which align well with the optical features.^41,43,44^

**Table 1:** Optical and Electrochemical Properties of CFB, CFB-Cl, CMB, and CMB-Cl

NFA	extinction coefficient (×10^5^ cm^–1^ M^–1^)^a^	λ_max_ [nm]		λ_onset_ (nm)^b^	*E*_g_^opt^ (eV)^c^	HOMO (eV)^d^	LUMO (eV)^d^	*E*_g_^ele^ (eV)^d^
		CF	film					
CFB	1.80	704	774	858	1.45	–5.85	–4.01	1.84
CFB-Cl	1.75	717	782	864	1.44	–5.88	–4.05	1.83
CMB	1.51	758	820	902	1.37	–5.69	–3.96	1.73
CMB-Cl	1.50	774	823	905	1.37	–5.71	–3.99	1.72

a Calculated in solution state.

b Determined in the film state.

c *E*_g_^opt^ = 1240/λ_onset_.

d Measured by cyclic voltammetry.

### 2.4 Computational Studies of Frontier Molecular Orbitals

Theoretical calculations were performed by Gaussian with B3LYP/6–311G­(d,p) as the basis set to obtain the electrostatic potential (ESP) as well as frontier molecular orbitals of CFB-Cl and CMB-Cl. To simplify the calculation process, all of the alkyl side chains were replaced by a methyl group. As shown in Figure 2e,f, negative ESP of CFB-Cl is distributed at central fluorine atoms, terminal chlorine atoms, and oxygen atoms, while positive ESP of CFB-Cl is mainly distributed at the alkyl side chains. As for CMB-Cl, the central methyl substituent leads to an extra positive ESP. As a result, the distribution of ESP is more imbalanced on the different sides of CMB-Cl, which further contributes to a larger dipole moment in the opposite direction with a value of 4.11 D compared to 0.73 D for CFB-Cl (Figure S3). Besides the ESP distribution, central fluorination and methylation also affect the frontier molecular orbitals of CFB-Cl and CMB-Cl. The donating nature of the methyl group leads to an upshifted HOMO and LUMO energy level of **–**5.79 and **–**3.80 eV compared to CFB-Cl, which has a HOMO/LUMO energy level of **–**5.99 and **–**3.90 eV (Figure S4), coinciding with the results obtained from *C–V* measurements.

### 2.5 Single-Crystal Analysis

To elucidate the molecular packing and intermolecular interactions of the NFAs, a single-crystal X-ray diffraction analysis was performed. A high-quality single crystal of CFB-Cl was successfully grown and analyzed, whereas the CMB-Cl crystal disintegrated severely before measurement, suggesting markedly different packing behaviors between the two molecules. The fragility of the CMB-Cl crystal may imply looser and less ordered packing relative to CFB-Cl. The crystallization procedure is described in the Supporting Information, and the detailed crystallographic parameters of CFB-Cl are summarized in Table S1. As shown in Figure S5, CFB-Cl crystallizes in the monoclinic space group *C*2/*c*, adopting a C-shaped molecular conformation. This conformation is stabilized by an intramolecular S···O interaction between the sulfur atom on the outer thiophene and the oxygen atom of the terminal group, which effectively locks the backbone geometry. CFB-Cl exhibits extremely small dihedral angles (<2°) between the outer thiophene and the terminal group, and an even smaller torsion angle of 0.25° within the o-BDP core, indicating exceptional molecular planarity that facilitates close intermolecular packing. Viewed along the *c*-axis (Figure 3a), CFB-Cl forms a kaleidoscopic 3D packing network characterized by highly ordered elliptical voids, accommodating four CFB-Cl molecules per unit cell. The side-view packing diagrams (Figure 3b,c) reveal three distinct dimeric packing motifs: CC–TT (core-to-core and terminal-to-terminal), CT–CT (core-to-terminal and core-to-terminal), and TT (terminal-to-terminal). According to previous studies,^55,56^ the coexistence of CC–TT and CT–CT modes enhances charge transport and suppresses charge recombination owing to the increased electronic coupling between neighboring molecules. Notably, in CT–CT mode, unique F···Cl halogen interactions are observed between the fluorinated o-BDP unit and the chlorinated terminal group, with an F–Cl distance of 3.58 Å. This short contact contributes to tighter molecular packing, accompanied by a short π–π stacking distance of 3.38 Å, which is expected to promote efficient charge transport within the solid state.

**Figure 3:**
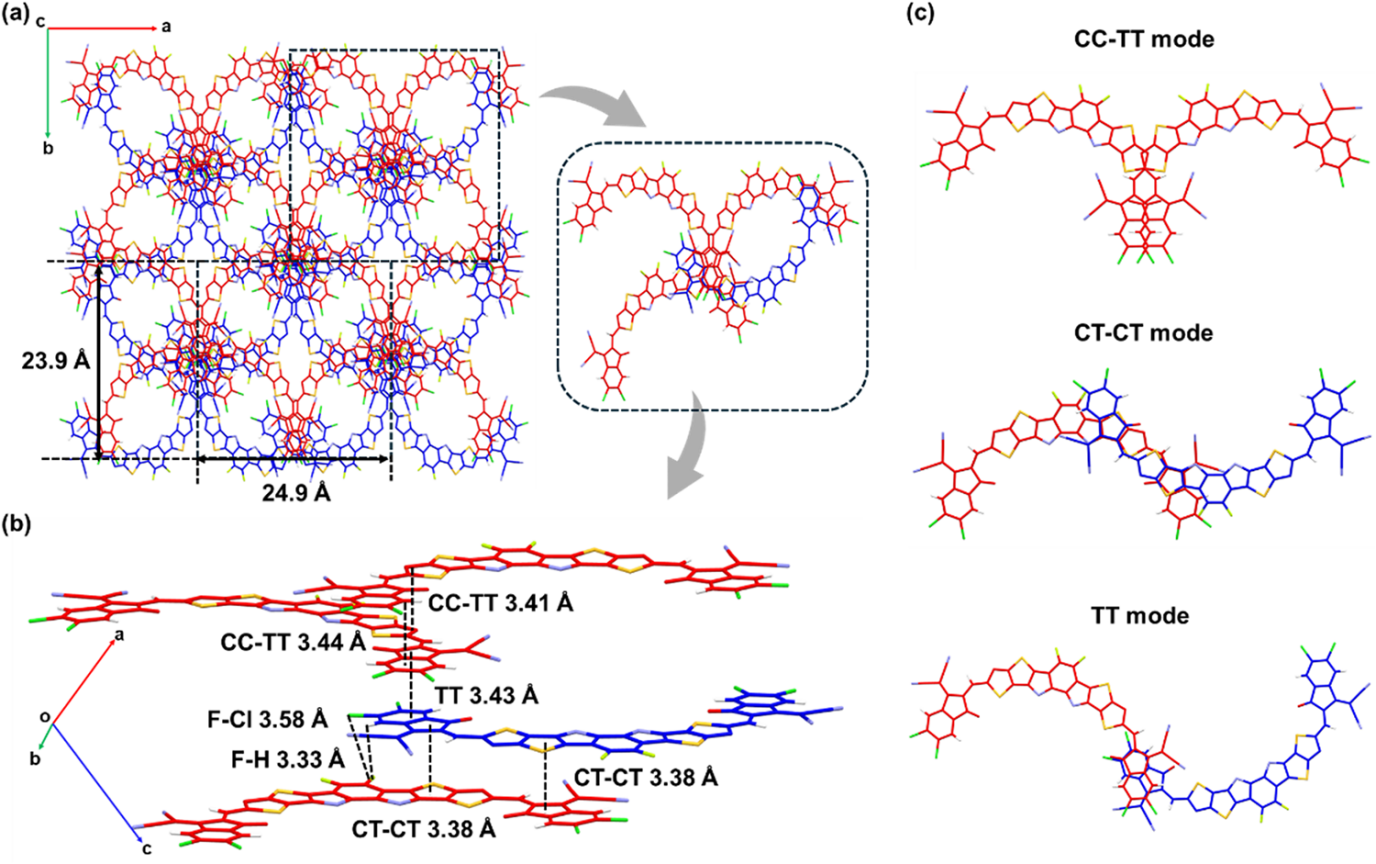
(a) 3D packing network of CFB-Cl viewed from the *c*-axis. (b) Side view of molecular packing in a unit cell of CFB-Cl. The type of packing modes and the corresponding π-π were denoted. (c) Three distinct dimeric packing modes that were observed in a unit cell of the CFB-Cl crystal.

### 2.6 Device Characteristics

To evaluate the photovoltaic performance, inverted organic solar cells with the configuration ITO/ZnO/PM6:NFA/MoO_3_/Ag were fabricated. The active layers were prepared by spin-coating the PM6:NFA blend solutions onto the ZnO electron-transporting layer. The optimizations of binary systems are summarized in Table S2. The current density–voltage (*J–V*) characteristics and external quantum efficiency (EQE) spectra of the optimized devices are shown in Figure 4a,b, and the corresponding photovoltaic parameters are summarized in Table 2. Benefiting from the compact molecular packing revealed by single-crystal analysis, the PM6:CFB-Cl device processed from o-xylene and thermally annealed at 160 °C exhibited an impressive PCE of 16.62%, with a *V*_oc_ of 0.859 V, a *J*_sc_ of 25.65 mA cm^–2^, and a high FF of 75.54%. In contrast, the PM6:CMB-Cl device, fabricated from chloroform and annealed at 140 °C, delivered a slightly lower PCE of 16.13%, with a higher *V*_oc_ of 0.904 V, *J*_sc_ of 26.29 mA cm^–2^, but a reduced FF of 68.04%. The differences in processing conditions and photovoltaic parameters indicate substantial morphological variations between the two binary blend films. To further enhance device performance, we incorporated CMB-Cl and CMB as secondary components in a PM6:CFB-Cl-based ternary device with various blend ratios to extend light absorption and improve photocurrent generation (Figure S6 and Table S3). Although CFB-Cl and CMB-Cl exhibit complementary absorption profiles, their incompatible processing solvents and limited miscibility might influence the morphology of the active layer. As a result, the PM6:CFB-Cl:CMB-Cl device yields a lower PCE of 16.31% with a decreased FF value. To mitigate this limitation, we replaced CMB-Cl with CMB containing fluorinated end groups, which offers better compatibility with *o*-xylene processing. The optimized PM6:CFB-Cl:CMB (1:0.6:0.6) device achieved a notable PCE of 17.26%, with an enhanced *V*_oc_ of 0.892 V, *J*_sc_ of 26.02 mA cm^–2^, and a well-maintained FF of 74.28%, underscoring the critical role of acceptor selection and blend compatibility in achieving high-performance ternary OPVs.

**Figure 4:**
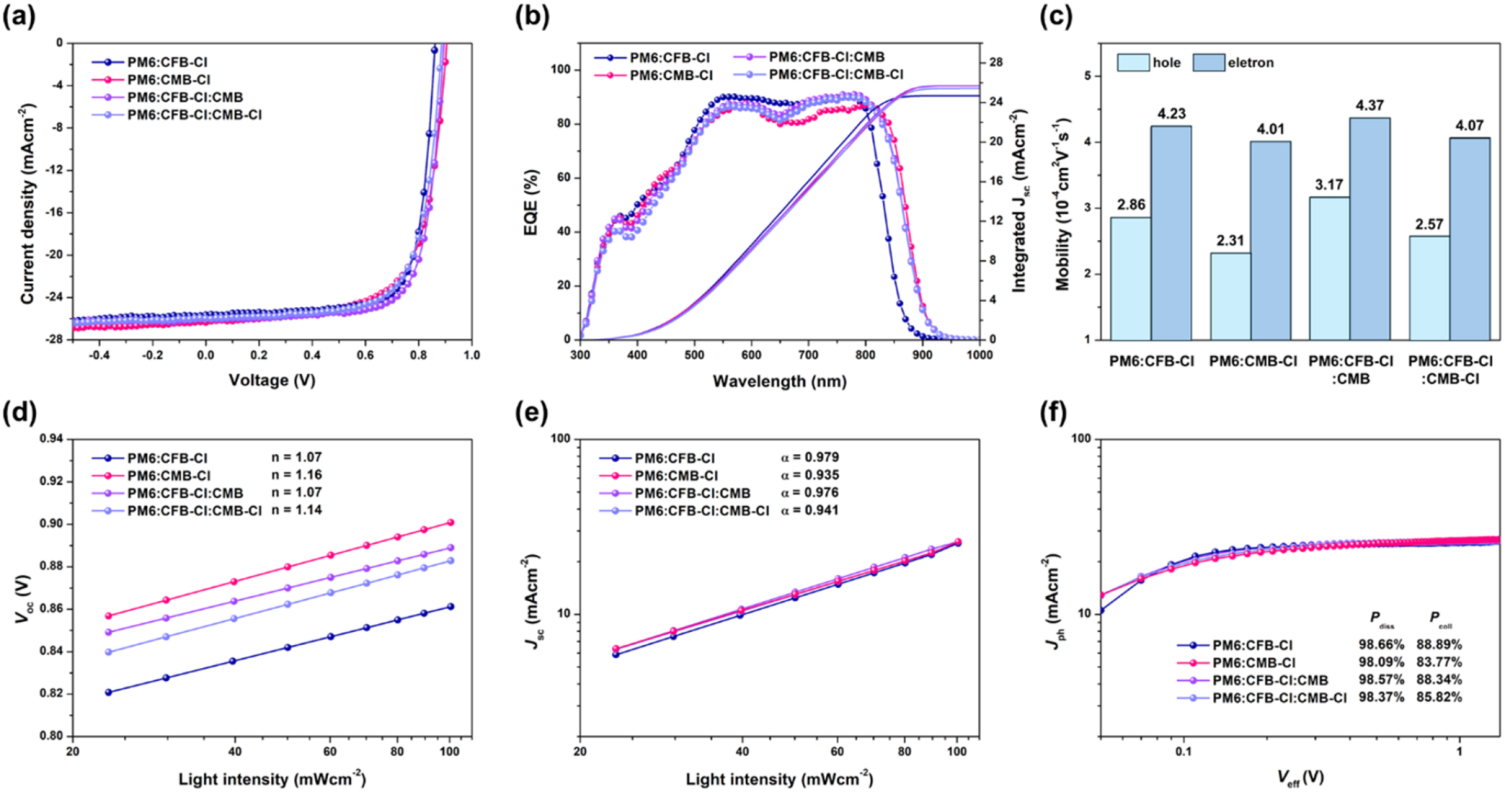
(a) *J–V* curves, (b) EQE spectra, (c) bar chart of Hole/electron mobilities, (d) *V*_oc_ versus light intensity plot, (e) *J*_sc_ versus light intensity plot, and (f) *J*_ph_ versus *V*_eff_ plot of optimized PM6:CFB-Cl, PM6:CMB-Cl, PM6:CFB-Cl:CMB, and PM6:CFB-Cl:CMB-Cl-based devices.

**Table 2:** Optimized Device Parameters of the PM6:CFB-Cl, PM6:CMB-Cl, PM6:CFB-Cl:CMB, and PM6:CFB-Cl:CMB-Cl-Based Devices

active layer	blend ratio in wt %	*V*_oc_ (V)	*J*_sc_ (mA cm^–2^)	FF (%)	PCE (%)
PM6:CFB-Cl^a^	1:1.2	0.859 (0.859 ± 0.003)	25.65 (25.68 ± 0.25)	75.54 (73.70 ± 1.45)	16.62 (16.22 ± 0.24)
PM6:CMB-Cl^b^	1:1.2	0.904 (0.904 ± 0.002)	26.29 (25.67 ± 0.40)	68.04 (68.37 ± 0.57)	16.13 (15.83 ± 0.21)
PM6:CFB-Cl:CMB^a^	1:0.6:0.6	0.892 (0.900 ± 0.004)	26.02 (25.94 ± 0.07)	74.28 (73.85 ± 0.34)	17.26 (17.18 ± 0.09)
PM6:CFB-Cl:CMB-Cl^a^	1:0.9:0.3	0.884 (0.882 ± 0.001)	25.97 (25.79 ± 0.21)	71.04 (70.24 ± 0.87)	16.31 (15.98 ± 0.26)

a Processed by *o*-xylene.

b Processed by chloroform.

### 2.7 Charge Transport and Recombination Dynamics

The electron (μ_e_) and hole (μ_h_) mobilities of the OPV devices were determined using the space-charge-limited current (SCLC) method.^57^ As illustrated in Figure 4c, The PM6:CFB-Cl and PM6:CFB-Cl:CMB devices exhibit higher and more balanced charge mobilities, with μ_e_/μ_h_ ratios of 1.48 and 1.38, respectively, indicating the formation of favorable and well-intermixed morphologies for charge transport. In contrast, the PM6:CMB-Cl and PM6:CFB-Cl:CMB-Cl devices display lower mobilities and more unbalanced μ_e_/μ_h_ ratios of 1.74 and 1.58, suggesting the presence of less favorable morphologies that impede charge transport.

To further probe the recombination processes, trap-assisted and bimolecular charge recombination were evaluated by the parameters n and α, respectively, following the relations *V*_oc_ ∝ *n*(*kT*/*q*) ln­(*P*_light_) and *J*_sc_ ∝ (*P*_light_)^α^.^57,58^ As shown in Figure 4d, the *n* values for PM6:CFB-Cl, PM6:CMB-Cl, PM6:CFB-Cl:CMB, and PM6:CFB-Cl:CMB-Cl are 1.07, 1.16, 1.07, and 1.14, respectively. The smaller nanovalues (≈1.07) of the PM6:CFB-Cl and PM6:CFB-Cl:CMB devices indicate minimal trap-assisted recombination, whereas the larger values for the other two devices suggest a greater density of trap states. In addition, the *J*_sc_–P_light_ plots (Figure 4e) yield α values of 0.979, 0.935, 0.976, and 0.941 for PM6:CFB-Cl, PM6:CMB-Cl, PM6:CFB-Cl:CMB, and PM6:CFB-Cl:CMB-Cl blends, respectively. The α values approaching unity for PM6:CFB-Cl and PM6:CFB-Cl:CMB confirm that bimolecular recombination is largely suppressed, consistent with their superior charge-transport balance. The relationship between photocurrent density (*J*_ph_) and effective voltage (*V*_eff_) was further examined to assess charge dissociation (*P*_diss_) and charge collection (*P*_coll_) efficiencies.^59,60^ As shown in Figure 4f, all devices exhibit similar *P*_diss_ values (98.09 and 98.66%), indicating nearly complete exciton dissociation. However, *P*_coll_ values of 88% for PM6:CFB-Cl and PM6:CFB-Cl:CMB are significantly higher than those for PM6:CMB-Cl and PM6:CFB-Cl:CMB-Cl (83 and 85%, respectively), demonstrating more efficient charge collection in the former systems.

To gain deeper insight into carrier dynamics, transient photocurrent (TPC) and transient photovoltage (TPV) measurements were performed to determine charge extraction times and carrier lifetimes, respectively (Figure S7).^61,62^ As summarized in Table S4, PM6:CFB-Cl and PM6:CFB-Cl:CMB exhibit faster charge extraction times (0.36 μs) than PM6:CMB-Cl (0.45 μs) and PM6:CFB-Cl:CMB-Cl (0.42 μs). Moreover, both show prolonged carrier lifetimes exceeding 44 μs, in contrast to 33 and 35 μs for PM6:CMB-Cl and PM6:CFB-Cl:CMB-Cl, respectively. Collectively, these results demonstrate that the fluorinated *o*-BDP unit can improve the suppressed charge recombination, balanced carrier transport, rapid charge extraction, and extended carrier lifetimes in a binary device. In a ternary device, incorporating CMB into the PM6:CFB-Cl system enhances the charge mobility and carrier lifetime.

### 2.8 Contact Angle Measurements and Miscibility

To elucidate the difference in the interaction between the two materials in the binary and ternary devices, contact angle measurements were conducted to evaluate the miscibility among the blend components. The experimental droplet images are shown in Figure S8, and the corresponding calculated surface energies are summarized in Table S5. The miscibility between two materials can be quantitatively assessed using the Flory–Huggins interaction parameter (χ), which is calculated according to the equation: $\chi =k{\left(\sqrt{\gamma_{1}}-\sqrt{\gamma_{2}}\right)}^{2}$ , where *k* is a constant and *γ*_1_ and *γ*_2_ are the surface energies of the two materials. As shown in Table S5, the χ value between PM6 and CFB-Cl (0.35 *k*) is significantly smaller than that between PM6 and CMB-Cl (0.83 *k*), indicating better miscibility between PM6 and CFB-Cl. In addition, the χ value between CFB-Cl and CMB (0.02 *k*) is markedly lower than that of CFB-Cl and CMB-Cl (0.10 *k*), demonstrating the formation of an alloy-like domain between CFB-Cl and CMB. These results clearly suggest that the superior miscibility of CFB-Cl with both PM6 and CMB contributes to the favorable film morphology and consequently to the higher FF observed in the PM6:CFB-Cl and PM6:CFB-Cl:CMB devices.

### 2.9 Device Film Morphology Analysis

The molecular packing and morphology of the neat and blend films were investigated by using grazing-incidence wide-angle X-ray scattering (GIWAXS). The corresponding 2D diffraction patterns and 1D line-cut profiles along the *q*_*xy*_ and *q*_*z*_ directions are presented in Figure 5. The structural parameters are listed in Table 3. As shown in Figure 5a, the neat CFB-Cl and CMB-Cl films exhibit similar diffraction features, characterized by a strong broad (010) π–π stacking peak in the *q*_*z*_ direction at 1.78 and 1.79 Å^–1^, corresponding to π–π stacking distance (*d*_π_) of 3.53 and 3.51 Å, respectively, and a relatively weak (100) reflection in the *q*_*xy*_ direction at 0.38 and 0.37 Å^–1^, corresponding to lamellar distance (*d*_l_) of 16.53 and 16.98 Å. These observations indicate a predominant face-on orientation for both materials. The PM6:CFB-Cl blend film retains this face-on orientation, exhibiting a prominent (010) reflection at *q*_*z*_ = 1.78 Å^–1^ and a clear (100) peak at *q*_*xy*_ = 0.31 Å^–1^. The presence of only a single set of (010) and (100) peaks suggests suppressed self-aggregation of the individual components and good miscibility between PM6 and CFB-Cl, consistent with the high fill factor (FF) and low χ values observed for the corresponding devices. In contrast, the PM6:CMB-Cl film also shows a face-on orientation with a (010) peak at *q*_*z*_ = 1.79 Å^–1^, but two distinct (100) reflections are observed in the in-plane direction at 0.30 and 0.36 Å^–1^, corresponding to the lamellar stacking for PM6 and CMB-Cl, respectively. These separate diffraction signals indicate phase segregation and the formation of distinct PM6 and CMB-Cl domains, consistent with the relatively large Flory–Huggins interaction parameter (χ). Upon introduction of CMB into the PM6:CFB-Cl system, the *d*_π_ increases to 3.55 Å. However, the higher π–π stacking coherence length (*L*_c π–π_) values in PM6:CFB-Cl:CMB (26.30 Å) compared with those of PM6:CFB-Cl (25.70 Å) indicate a larger π-π stacking domain size in the ternary device, which facilitates charge transport. In addition, the presence of only a single (010) and (100) peak indicates that the PM6:CFB-Cl:CMB film maintains a well-mixed morphology with ordered molecular packing. In contrast, incorporation of CMB-Cl into the PM6:CFB-Cl blend leads to an enlarged *d*_π_ and reduced *L*_c π–π_, reflecting looser and less ordered packing. Consequently, the PM6:CFB-Cl:CMB devices exhibit a significantly higher FF compared with the PM6:CFB-Cl:CMB-Cl counterparts.

**Figure 5:**
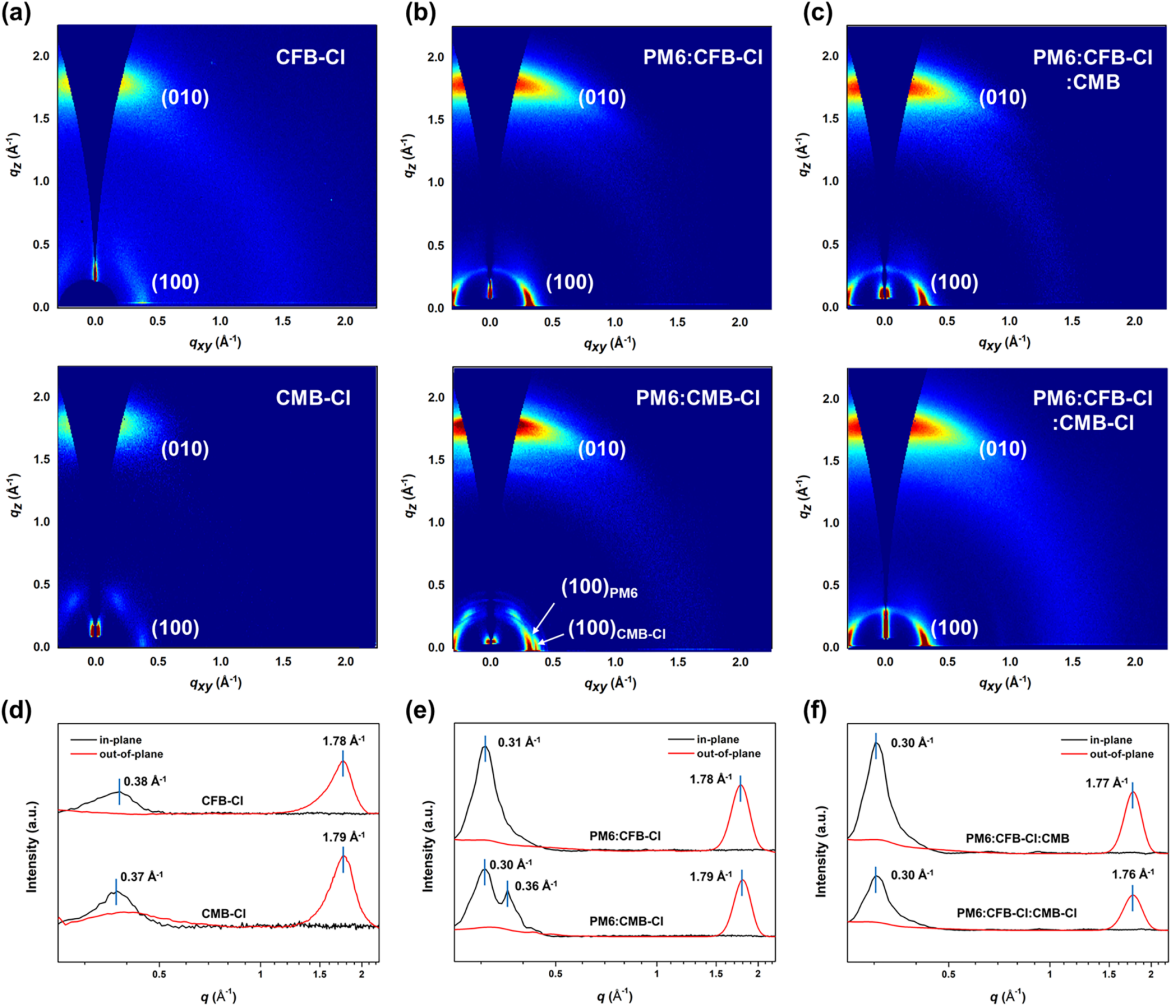
2D GIWAXS patterns of (a) neat NFA films, (b) binary blended films, and (c) ternary blended films. The corresponding 1D profiles along the *q*_*xy*_ and *q*_*z*_ directions of (d) neat NFA films, (e) binary blended films, and (f) ternary blended films.

**Table 3:** Parameters Derived from the GIWAXS Patterns of the Neat Films and the Blend Films, Including the Out-of-Plane and In-Plane Reflection Peaks Centered at *q*_*z*_ and *q*_*xy*_ Direction, with the Corresponding *d*-Spacing and the Correlation Length *L*_c_, Which Is Deduced from the Full Width at Half-Maximum (fwhm) Using the Scherrer Equation with Shape Factor 0.9

	in-plane reflection peak				out-of-plane reflection peak			
film	*q*_*xy*_ (Å^–1^)	*d*_l_ (Å)^a^	fwhm (Å^–1^)	*L*_c l_ (Å)^b^	*q*_*z*_ (Å^–1^)	*d*_π_ (Å)^c^	fwhm (Å^–1^)	*L*_c π–π_ (Å)^d^
CMB-Cl	0.37	16.98	0.115	49.17	1.79	3.51	0.333	16.98
CFB-Cl	0.38	16.53	0.125	45.24	1.78	3.53	0.309	18.30
PM6:CMB-Cl	0.30	20.94	0.048	117.81	1.79	3.51	0.207	27.31
	0.36	17.45	0.045	125.66				
PM6:CFB-Cl	0.31	20.27	0.057	99.21	1.78	3.53	0.220	25.70
PM6:CFB-Cl:CMB	0.30	20.94	0.058	97.50	1.77	3.55	0.215	26.30
PM6:CFB-Cl:CMB-Cl	0.30	20.94	0.059	95.85	1.76	3.57	0.226	25.02

a Lamellar distance.

b Coherence length of lamellar stacking.

c π–π stacking distance.

d Coherence length of π–π stacking.

The surface morphology of the neat NFAs and blend films was examined by using atomic force microscopy (AFM). As shown in Figure 6, both CFB-Cl and CMB-Cl neat films form smooth and uniform surfaces with root-mean-square (RMS) roughness values of approximately 0.5 nm. However, the binary blend films exhibit markedly different morphologies. The PM6:CFB-Cl film shows a well-defined fibrillar network with a uniform surface texture and an RMS roughness of 2.09 nm, indicative of an optimized nanoscale phase separation beneficial for charge transport. In contrast, the PM6:CMB-Cl film displays a much rougher surface (RMS = 3.41 nm) and lacks any discernible fibrillar features, suggesting pronounced phase separation and self-aggregation between the donor and acceptor components. In the ternary blend systems, the PM6:CFB-Cl:CMB film exhibits a slightly increased RMS of 2.27 nm, whereas the PM6:CFB-Cl:CMB-Cl film shows a marginally lower RMS of 1.98 nm. These variations are consistent with the trend observed in the GIWAXS analysis of the *L*_c_(π–π) coherence length. Collectively, these results highlight that the well-mixed, ordered, and uniform morphology of the PM6:CFB-Cl and PM6:CFB-Cl:CMB films plays a crucial role in achieving the superior FF and overall device performance compared to the other systems.

**Figure 6:**
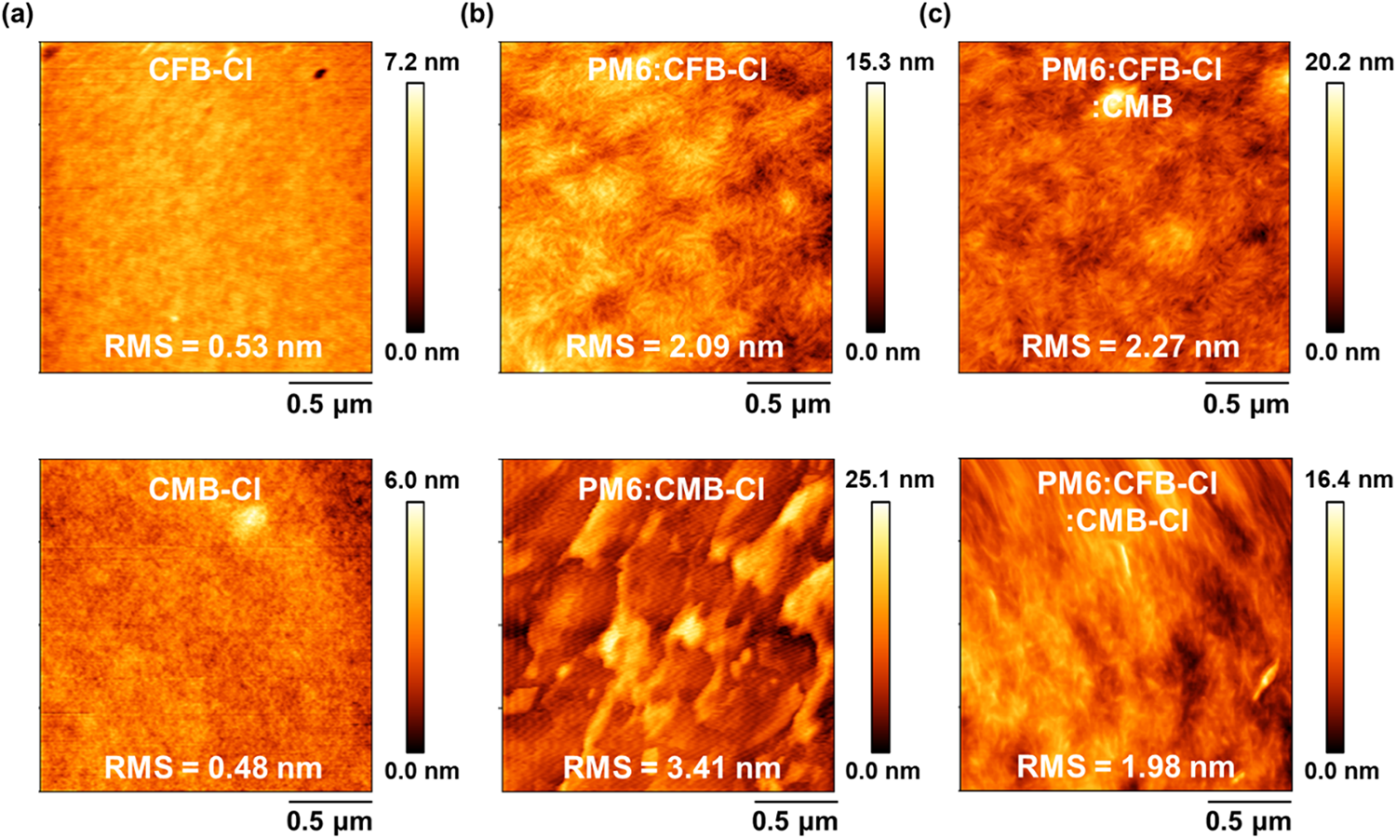
AFM images of (a) CFB-Cl and CMB-Cl neat films, (b) PM6:CFB-Cl and PM6:CMB-Cl binary films, and (c) PM6:CFB-Cl:CMB and PM6:CFB-Cl:CMB-Cl ternary films.

## 3 Conclusions

The strategic incorporation of chlorinated end groups into fluorinated and methylated o-BDP cores yielded two novel NFAs, CFB-Cl and CMB-Cl. Compared to their terminal-fluorinated counterparts, both NFAs exhibit red-shifted absorption, higher melting points, and stronger intermolecular interactions, attributed to the introduction of chlorinated end groups. The CFB-Cl crystal exhibited a compact 3D network stabilized by F···Cl interactions, while CMB-Cl formed fragile crystals, reflecting distinct packing characteristics, demonstrating the critical role of halogen–halogen interactions in determining solid-state organization and device performance. These structural differences translated directly into film morphologies and device efficiencies, with PM6:CFB-Cl achieving superior FF (75.54%) and PCE (16.62%) compared to PM6:CMB-Cl. Guided by morphological and miscibility analyses, the ternary PM6:CFB-Cl:CMB system was optimized to exploit complementary absorption and enhanced compatibility, resulting in the highest PCE of 17.26% with excellent charge-transport balance and minimized recombination losses. Overall, this work demonstrates that synergistic fluorine–chlorine interactions, coupled with rational acceptor selection, provide an effective molecular-design pathway for tailoring molecular packing and achieving high-performance o-BDP-based NFAs and efficient ternary organic solar cells.

## 4 Experimental Section

### 4.1 Synthesis of CFB-Cl

To a solution of compound **3a** (36.9 mg, 0.03 mmol) and 2-(5,6-dichloro-3-oxo-2,3-dihydro-1H-inded-1-ylidene)­malononitrile (34.2 mg, 0.13 mmol) in chloroform/DMF (3.3 mL/0.7 mL, v/v) was added trimethylsilyl chloride (0.84 mL) dropwise. The resulting blue solution was stirred at 50 °C for 16 h. After cooling to room temperature, the mixture was extracted with dichloromethane and water. The collected organic layer was dried over anhydrous MgSO_4_. After the removal of the solvent under reduced pressure, the crude product was purified by column chromatography on silica gel (hexane/dichloromethane, v/v, 2/1) to get the blue solid **CFB-Cl** (47.5 mg, 92%). ^1^H NMR (400 MHz, CDCl_3_): δ 9.19 (s, 2H), 8.81 (s, 2H), 7.96 (s, 2H), 4.66 (d, *J =* 7.6 Hz, 4H), 3.21 (t, *J =* 7.5 Hz, 4H), 2.06 (m, 2H), 1.87–1.83 (m, 4H), 1.51–1.47 (m, 4H), 1.44–1.21 (m, 28H), 1.20–0.93 (m, 40H), 0.90–0.77 (m, 20H), 0.73 (t, *J =* 7.1 Hz, 6H). ^13^C NMR (100 MHz, CDCl_3_): δ 186.24, 158.84, 154.02, 145.37, 139.81, 139.25, 138.77, 138.02, 136.29, 136.10, 135.97, 133.73, 129.22, 127.05, 126.92, 125.08, 120.28, 115.09, 114.57, 114.37, 77.34, 77.09, 76.83, 68.05, 55.40, 38.91, 31.99, 31.95, 31.69, 31.37, 29.86, 29.72, 29.69, 29.56, 29.49, 29.46, 29.42, 29.32, 25.69, 22.76, 22.71, 22.62, 14.18, 14.13. ^19^F NMR (376 MHz, CDCl_3_): −154.58(s); HRMS (FD, C_98_H_118_N_6_O_2_F_2_S_4_Cl_4_): calcd 1716.6898; found 1716.6916.

### 4.2 Synthesis of CMB-Cl

To a solution of compound **3b** (36.7 mg, 0.03 mmol) and 2-(5,6-dichloro-3-oxo-2,3-dihydro-1*H*-inded-1-ylidene)­malononitrile (34.2 mg, 0.13 mmol) in chloroform/DMF (3.3 mL/0.7 mL, v/v) was added trimethylsilyl chloride (0.84 mL) dropwise. The resulting blue solution was stirred at 50 °C for 16 h. After cooling to room temperature, the mixture was extracted with dichloromethane and water. The collected organic layer was dried over anhydrous MgSO_4_. After removing the solvent under reduced pressure, the crude product was purified by column chromatography on silica gel (hexane/dichloromethane, v/v, 2/1) to get the blue solid **CMB-Cl** (45.7 mg, 89%). ^1^H NMR (400 MHz, CDCl_3_): δ 9.15 (s, 2H), 8.78 (s, 2H), 7.93 (t, 2H), 4.62 (d, *J =* 8.1 Hz, 4H), 3.20 (t, *J =* 7.5 Hz, 4H), 2.71 (s, 6H), 2.05–1.95 (m, 2H), 1.90–1.80 (m, 4H), 1.51–1.45 (m, 4H), 1.40–1.22 (m, 30H), 1.17–0.82 (m, 40H), 0.81–0.67 (m, 24H). ^13^C NMR (100 MHz, CDCl_3_): δ 186.07, 158.86, 154.06, 144.68, 139.21, 138.87, 138.51, 137.14, 136.07, 135.69, 133.47, 131.87, 131.27, 126.76, 125.14, 124.79, 122.52, 119.14, 115.26, 114.78, 77.31, 76.99, 76.68, 67.96, 67.80, 54.94, 38.77, 31.90, 31.61, 31.33, 30.54, 29.81, 29.79, 29.74, 29.66, 29.62, 29.50, 29.46, 29.42, 29.39, 29.34, 29.28, 25.60, 25.55, 25.47, 22.67, 22.64, 22.56, 15.89, 14.12, 14.10, 14.09; HRMS (FD, C_100_H_124_N_6_O_2_S_4_Cl_4_): calcd 1708.7423; found 1708.7417.

### 4.3 Device Fabrication

All of the devices were fabricated using the following procedures: The ITO-coated glass was treated with UV-ozone for 25 min, spin-coated with ZnO solution (preheated at 45 °C for 30 min), and then baked at 180 °C for 20 min. The PM6:NFA blends were dissolved in chloroform and *o*-xylene and then stirred for 1 h at 45 °C and 20 h at 60 °C, respectively. The blend solutions were spin-coated on top of the ZnO layer as the active layer. The resulting substrates were thermally annealed for 10 min at different temperatures, followed by the sequential formation of a MoO_3_ layer (7 nm) and a silver anode (150 nm) by thermal vapor deposition at a pressure below 1.5 × 10^–6^ Torr. The devices without encapsulation were characterized immediately in ambient conditions. Current–voltage characteristics were measured by a Keithley 2400 SMU under the irradiation of an AM 1.5G San-Yi solar simulator with a JIS AAA spectrum. EQE spectra were measured in ambient conditions using a lock-in amplifier with a current preamplifier under short-circuit conditions with illumination by monochromatic light from a 250 W quartz-halogen lamp (Osram) passing through a monochromator (Spectral Products CM110).

## Supplementary Material



## Notes

Materials, Instruments and characterization, TGA measurement results, cyclic voltammetry (*C–V*) characteristics, density functional theory (DFT) calculation of frontier molecular orbitals, single-crystal growth and crystallographic data, device optimization, space-charge limited current (SCLC) characteristics, transient photocurrent and transient photovoltage measurements, GIWAXS measurements, ^1^H and ^13^C NMR spectra, and references (PDF)
