# Supplementary material for: Fluorinated Benzodipyrrole-Based Non-Fullerene Acceptors with Chlorinated End Groups Exhibiting Fluorine–Chlorine Interactions for Suppressed Charge Recombination in Organic Photovoltaics
Source: ACS Appl Mater Interfaces. 2026 Feb 5;18(6):10138–48. doi: 10.1021/acsami.5c23292 (PMC12926939; doi:10.1021/acsami.5c23292)
Supplement: Supplementary file 1 [file am5c23292_si_002.pdf]

Supporting Information (SI)

Fluorinated Benzodipyrrole-Based Non-Fullerene Acceptors with Chlorinated End-Groups Exhibiting Fluorine–Chlorine Interactions for Suppressed Charge Recombination in Organic Photovoltaics

Yan-Bo Wang,[a] Yung-Jing Xue,[a] Hong-Yi Chen,[a] Chia-Lin Tsai,[a] Jin Lee,[a] Bing-Huang Jiang,[c] Chih-Ping Chen,[c] Fang-Chung Chen,[d] Chain-Shu Hsu,[a,b] Ta-Ya Chu,[e] Jianping Lu,[e] Su-Ying Chien,[f] and Yen-Ju Cheng*[a,b]

[a]*Department of Applied Chemistry, National Yang Ming Chiao Tung University, 1001 University Road, Hsinchu, 30010, Taiwan.*

[b]*Center for Emergent Functional Matter Science, National Yang Ming Chiao Tung University, 1001 University Road, Hsinchu, 30010, Taiwan*

[c]*Department of Materials Engineering and Organic Electronics Research Center, Ming Chi University of Technology, New Taipei City 24301, Taiwan*

[d]*Department of Photonics, National Yang Ming Chiao Tung University, 1001 University Road, Hsinchu, 30010, Taiwan.*

[e]*Quantum and Nanotechnologies Research Centre, National Research Council of Canada, 1200 Montreal Road, Ottawa, ON, Canada.*

[f]*Instrumentation Center, National Taiwan University, No.1, Sec. 4, Roosevelt Road, Taipei 10617, Taiwan.*

Email: yjcheng@nycu.edu.tw

Table of contents

1. Materials, Instruments and characterization......S2

2. TGA measurement results S2

3. Cyclic voltammetry (CV) characteristics......S3

4. Density functional theory (DFT) calculation of frontier molecular orbitals S3

5. Single crystal growth and crystallographic data......S4

6. Device optimization......S6

7. Space-charge limited current (SCLC) characteristics......S7

8. Transient photocurrent and Transient photovoltage measurements S8

10. GIWAXS measurements S9

11. ^{1}H and ^{13}C NMR spectra S11

12. References......S16

1. Materials, Instruments and characterization

All reagents and chemicals were purchased from commercial sources and were used without further purification unless noted otherwise. PM6 was purchased from Solarmer, Inc. Varian-400 MHz, JEOL-400 MHz and JEOL-500 MHz instrument spectrometers were used to measure ^{1}H, ^{13}C and ^{19}F spectrums. Deuterated chloroform ($CDCl_3$) with TMS as internal were used as references in NMR measurements. The mass spectra of the samples were recorded on JEOL T200-GC high resolution spectrometer using field desorption (FD) method. UV-vis absorption spectra were measured on HP8453 UV-vis spectrophotometer. Differential scanning calorimetry (DSC) and thermogravimetric analysis (TGA) were conducted on a TA Q200 Instrument and a TA TGA55 Instrument under nitrogen atmosphere at heating/cooling rate of 10 °C/min. Surface topography was investigated using Veeco diInnova AFM and standard tips (Tapping mode; L: 240 μm; Resonance Frequency: 70 kHz; Spring Constant: 2 N/m).

2. TGA measurement results

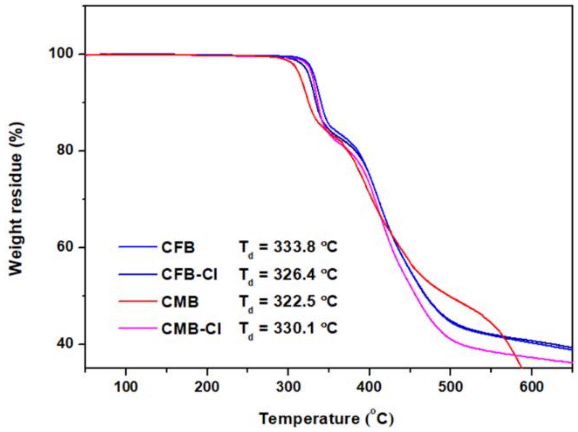

Figure S1. The results of the TGA measurement of CFB, CFB-Cl, CMB and CMB-Cl.

3. Cyclic voltammetry (CV) characteristics

CV was measured on a CH instruments electrochemical analyzer. A carbon glass was used as the working electrode and an Ag/AgCl electrode as the reference electrode. Solution of 0.1 M tetrabutylammonium hexafluorophosphate in acetonitrile was used as electrolyte. Ferrocence, whose HOMO energy level is −4.8 eV with respect to zero vacuum level, was used as standard to calibrate the CV curves. The HOMO and LUMO energy levels were obtained from the following equation:

$$E_{HOMO} = -|E_{ox}^{onset} - E_{ferrocene}^{onset} + 4.8|\ \text{eV} \qquad \text{(S1)}$$

$$E_{LUMO} = -|E_{red}^{onset} - E_{ferrocene}^{onset} + 4.8|\ \text{eV} \qquad \text{(S2)}$$

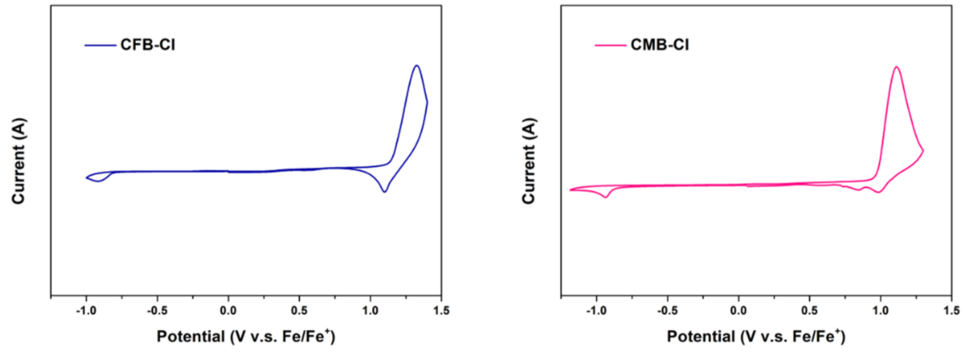

Figure S2. Cyclic voltammogram of CFB-Cl and CMB-Cl.

4. Density functional theory (DFT) calculation of frontier molecular orbitals

DFT calculation was conducted by Gaussian 09 suite15 at the B3LYP/6-311G(d,p) level. All alkyl side chains were simplified to methyl group in the computational model compounds.

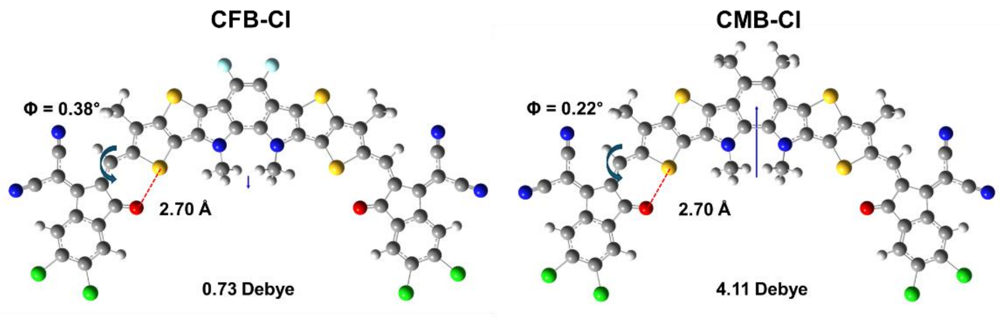

Figure S3. Top views of optimized structure of CFB-Cl and CMB-Cl. Dipole moment, S-O distance and torsion angle (*Φ*) were also denoted. The atomic species C is labeled by gray, N by blue, O by red, F by cyan, Cl by green and S by yellow.

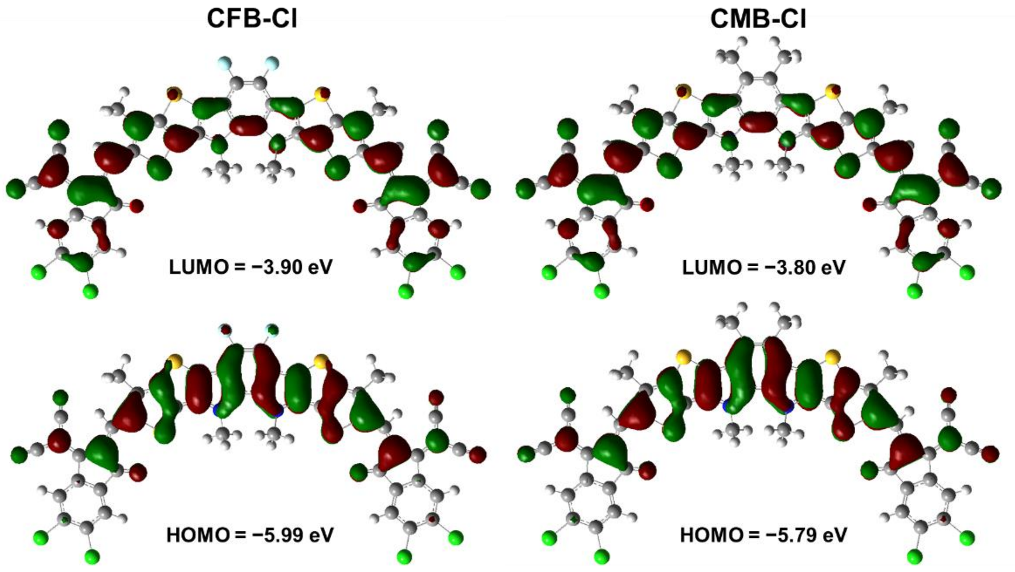

Figure S4. HOMO/LUMO distribution of CFB-Cl and CMB-Cl.

5. Single crystal growth and crystallographic data

5.1 Single crystal growth

Solutions of 1.6 mg of CFB-Cl and CMB-Cl dissolved in 2.0 mL of chloroform were separately prepared in 4 mL vials. The vials were capped by aluminum foil with a hole punched using a 18G needle in the middle for vapor diffusion. The capped 4 mL vials were separately transferred into 20 mL vials which contained 2.5 mL of acetonitrile. The resulting vials were tightly sealed by parafilm, stored in dark and left standing. After one to two weeks, the crystals would form in each vial. Unfortunately, the crystals of CMB-Cl were extremely fragile. The crystals of CMB-Cl cracked as soon as we opened the 20 mL vial. As a result, only the crystals of CFB-Cl could be further analyzed.

5.2 Crystallographic data

The single crystal of CFB-Cl was mounted on a CryoLoop with Parabar 10312 oil for the single-

crystal X-ray diffraction experiments at −73 °C. The crystal size is 0.602 × 0.233 × 0.146 mm^3. The single-crystal X-ray diffraction data was collected in-house on a Bruker D8 Venture diffractometer equipped with a Mo-target (Kα = 0.71073 Å) microfocus X-ray generators and a PHOTON-II CMOS detector. The temperature was adjusted with a nitrogen flow (Oxford Cryosystems, 800+ series). After collection, the cell refinement and data integration were carried out by Bruker SAINT software package using a narrow-frame algorithm and were corrected for absorption effects using the Multi-Scan method (SADABS).[1] Moreover, the molecular structure was solved by SHELXT (Sheldrick 2015) and refined by SHELXL-2019/1 (Sheldrick, 2019).[2] The final anisotropic full-matrix least-squares method was used to refine on F^2 with variables parameters to determine crystal structure. All calculations were performed using the APEX4[3] software package. The crystallographic data of CFB-Cl are listed in Table S1.

Table S1. Crystallographic data of CFB-Cl.

CCDC number	2482333
Empirical formula	$C_{98}H_{118}N_6O_2F_2S_4Cl_4$
Formula weight	1720.02
Temperature	200(2) K
Wavelength	0.71073 Å
Crystal system	Monoclinic
Space group	C2/c
Unit cell dimensions	a = 24.5501(7) Å α = 90°<br>b = 23.4652(6) Å β = 107.7585(9)°<br>c = 34.4330(9) Å γ = 90°
Volume	18890.7(9) $Å^3$
Z	8
Density (calculated)	1.210 Mg/m^3
Absorption coefficient	0.268 mm^{-1}
F(000)	7312
Crystal size	0.602 × 0.233 × 0.146 mm^3
θ range for data collection	1.927 to 26.250°
Index ranges	−26 <= h < = 30, −29 <= k <= 29, −42 <= l <= 42
Reflections collected	61324
Independent reflections	19047 [R(int) = 0.0441]
Completeness to θ = 25.000°	99.8%
Refinement method	Full-matrix least-squares on F^2
Data/restraints/parameters	19047/493/1003
Goodness-of-fit on F^2	1.451
Final R indices [I>2sigma(I)]	R1 = 0.1224, wR2 = 0.3560
R indices (all data)	R1 = 0.1693, wR2 = 0.4058
Largest diff. peak and hole	1.175 and −1.122 e.$Å^{-3}$

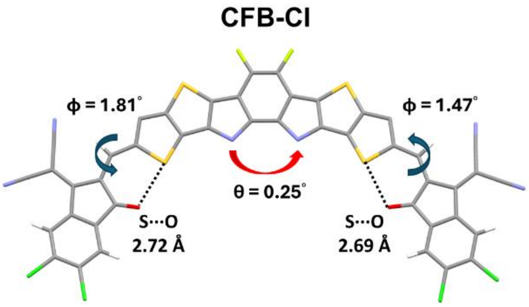

Figure S5. The monomolecular crystallographic structure of CFB-Cl with the dihedral angle (Φ) and torsion (θ) angle denoted.

6. Device optimization

Table S2. Optimization of PM6:CFB-Cl and PM6:CMB-Cl binary devices. (PM6:NFA = 1:1.2)

Active layer	Processing solvent	Annealing temperature [°C]	V_{oc} [V]	J_{sc} [mA cm^{-2}]	FF [%]	PCE [%]
PM6:CFB-Cl	chloroform	140	0.895	25.28	65.51	14.79
		150	0.891	24.56	67.74	14.90
		160	0.891	25.41	66.08	14.93
		170	0.89	24.69	65.81	14.43
	o-xylene	140	0.858	25.63	73.48	16.12
		150	0.860	25.30	74.86	16.25
		160	0.859	25.65	75.54	**16.62**
		170	0.858	25.14	71.75	15.44
PM6:CMB-Cl	chloroform	130	0.901	25.01	67.85	15.26
		140	0.904	26.29	68.04	**16.13**
		150	0.906	25.36	69.33	15.90
	o-xylene	130	0.879	22.19	67.16	13.07
		140	0.878	22.52	69.28	13.66
		150	0.887	22.04	68.00	13.25

Table S3. Optimization of PM6:CFB-Cl:CMB and PM6:CFB-Cl:CMB-Cl ternary devices.

Active layer	Processing solvent	Ratio (D:A1:A2)	Annealing temperature [°C]	V_{oc} [V]	J_{sc} [mA cm^{-2}]	FF [%]	PCE [%]

S
P
A
G
E

PM6:CFB-Cl :CMB	*o*-xylene	1:1.2:0	140	0.858	25.63	73.48	16.12
		1:0.9:0.3	140	0.892	25.83	73.02	16.83
		1:0.6:0.6	140	0.893	26.02	74.28	**17.26**
		1:0.3:0.9	140	0.896	26.01	70.87	16.52
		1:0:1.2	140	0.897	26.32	69.50	16.40
		1:0.6:0.6	150	0.891	25.81	74.04	17.03
PM6:CFB-Cl :CMB-Cl	*o*-xylene	1:1.2:0	140	0.858	25.63	73.48	16.12
		1:0.9:0.3	140	0.884	25.97	71.04	**16.31**
		1:0.6:0.6	140	0.883	25.56	69.86	15.73
		1:0.3:0.9	140	0.881	25.28	68.52	15.26
		1:0:1.2	140	0.878	22.52	69.28	13.66
		1:0.6:0.6	150	0.882	25.90	68.89	15.73
PM6:CFB-Cl :CMB-Cl	chloroform	1:1.2:0	140	0.895	25.28	65.51	14.79
		1:0.9:0.3	140	0.889	25.51	66.41	**15.06**
		1:0.6:0.6	140	0.886	25.55	66..32	14.79
		1:0.3:0.9	140	0.897	25.21	64.34	14.55
		1:0:1.2	140	0.904	26.29	68.04	16.13
		1:0.6:0.6	150	0.889	25.50	63.59	14.42

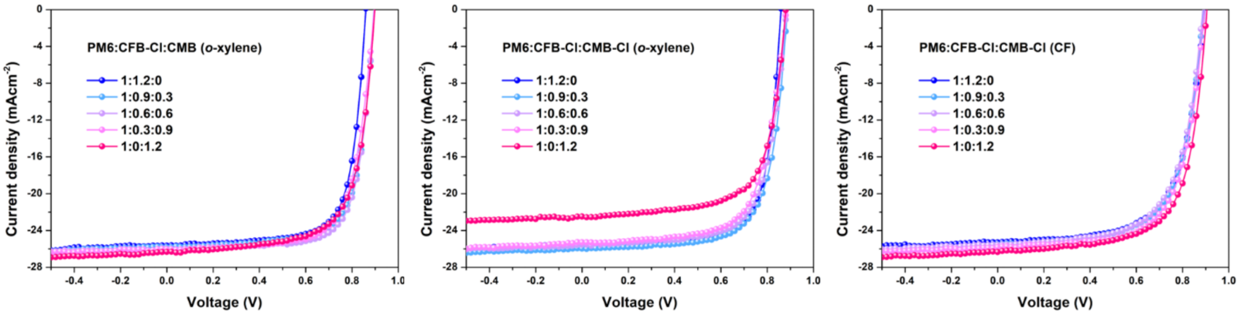

Figure S6. *J-V* curves of PM6:CFB-Cl:CMB and PM6:CFB-Cl:CMB-Cl devices under different D:A1:A2 ratio.

7. Space-charge limited current (SCLC) characteristics

The hole-only and electron-only devices were fabricated by employing the following device structure: ITO/PEDOT:PSS/active layer/Au for holes and ITO/ZnO/active layer/Al for electrons. The mobilities were obtained by taking current-voltage curves and fitting the results to the equation listed below:

$$J = \frac{9\varepsilon_0\varepsilon_r\mu V^2}{8L^3} \qquad \text{(S3)}$$

where J is the current density, ε_0 is the vacuum permittivity, ε_r is the relative dielectric constant, μ is the mobility, V is the voltage, and L is the film thickness.[4]

8. Transient photocurrent and Transient photovoltage measurements

Transient photocurrent (TPC) and Transient photovoltage (TPV) measurements were measured by the characterization platform Paios (Fluxim AG). The charge extraction time and carrier lifetime were extracted by the exponential decay fitting of the normalized TPC and TPV data, respectively.[5]

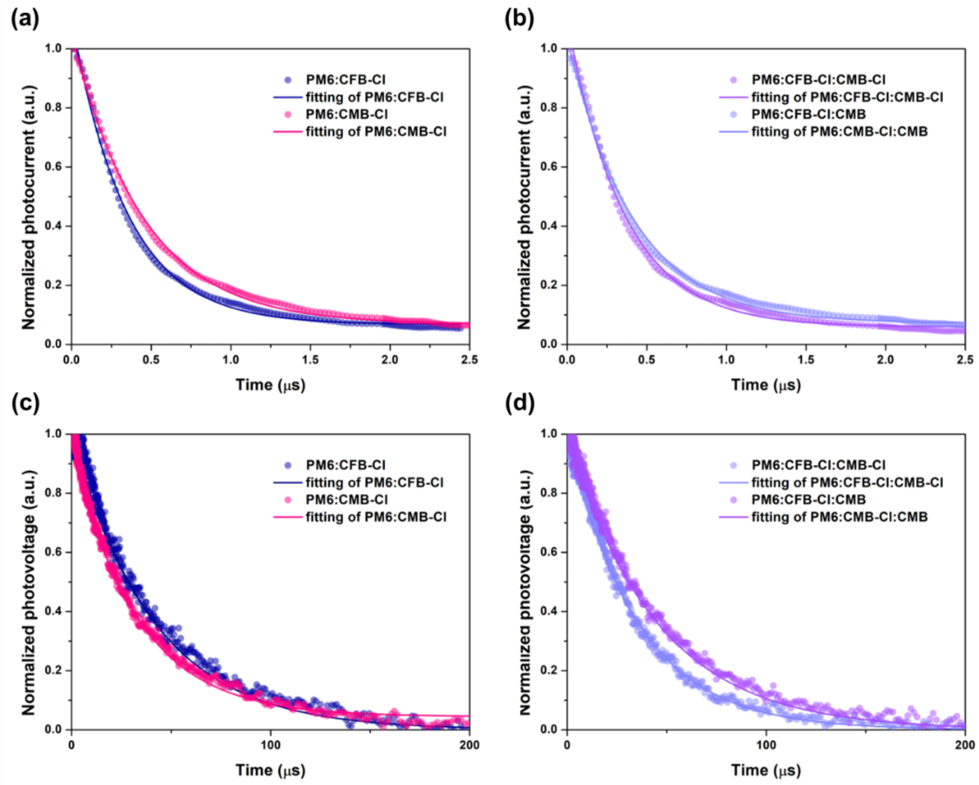

Figure S7. Transient photocurrent (TPC) results with their corresponding fitting curves (solid curves) of (a) PM6:CFB-Cl and PM6:CMB-Cl binary devices and (b) PM6:CFB-Cl:CMB and PM6:CFB-Cl:CMB-Cl ternary devices. Transient photovoltage (TPV) results with their corresponding fitting curves (solid curves) of (c) the binary devices and (d) the ternary devices.

Table S4. Charge extraction time (extracted from TPC) and carrier lifetime (extracted from TPv) of the OPVs.

blends	Charge extraction time [μs]	Carrier lifetime [μs]
PM6:CFB-Cl	0.36	44.23
PM6:CMB-Cl	0.45	32.73
PM6:CFB-Cl:CMB	0.36	44.59
PM6:CFB-Cl:CMB-Cl	0.42	35.08

9. Contact angle measurements

Contact angle measurements were performed by OCA25 with water and diethylene glycol (DEG) as test droplets. Flory-Huggins parameter between donor and acceptor ($\chi_{D\text{-}A}$) and that between CFB-Cl and other NFAs ($\chi_{CFB\text{-}Cl\text{-}A}$) were obtained from the following equation:

$$\chi_{D-A} = \left(\sqrt{\gamma_{PM6}} - \sqrt{\gamma_{NFA}}\right)^2 \quad \text{(S4)}$$

$$\chi_{CFB-Cl-A} = \left(\sqrt{\gamma_{CFB-Cl}} - \sqrt{\gamma_{NFA}}\right)^2 \quad \text{(S5)}$$

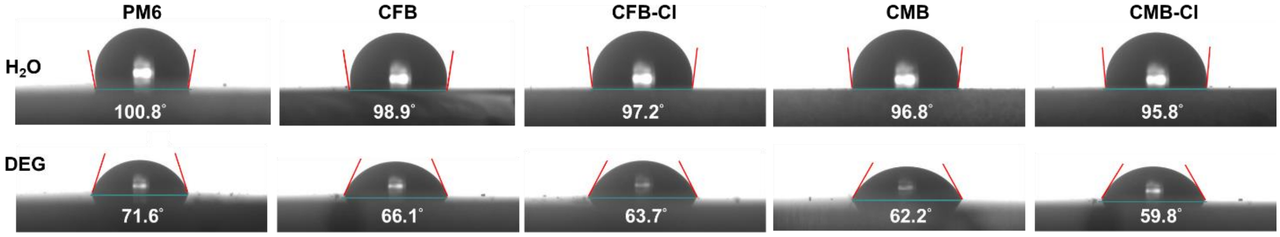

Figure S8. Contact angle measurement results of neat film of PM6 and the NFAs.

Table S5. Contact angle, surface energy and Flory-Huggins parameter of PM6 and the NFAs.

film	H_2O [°]	DEG [°]	γ [a)] [mN/m]	$\chi_{D\text{-}A}$ [b)] [a.u.]	$\chi_{CFB\text{-}Cl\text{-}A}$ [c)] [a.u.]
PM6	100.8	71.6	24.91	-	-
CFB	98.9	66.1	30.09	0.24 K	-
CFB-Cl	97.2	63.7	31.22	0.36 K	-
CMB	96.8	62.2	32.81	0.54 K	0.02 K
**CMB-Cl**	**95.8**	**59.8**	**34.87**	**0.84** K	0.10 K

[a)]Surface tension of the neat film; [b)]Flory-Huggins interactive parameters between PM6 and NFA. $\chi_{D-A} = \left(\sqrt{\gamma_{PM6}} - \sqrt{\gamma_{NFA}}\right)^2$, where K is a constant; [c)]Flory-Huggins interactive parameters between CFB-Cl and other NFA. $\chi_{CFB-Cl-A} = \left(\sqrt{\gamma_{CFB-Cl}} - \sqrt{\gamma_{NFA}}\right)^2$, where K is a constant.

10. GIWAXS measurements

The 25A1 coherent X-ray scattering beamline of the Taiwan Photon Source (TPS) was used to perform Grazing incidence wide-angle X-ray scattering (GIWAXS) measurements of the neat and blended films. The 2D GIWAXS patterns of the test film were collected with an Eiger X 1M detector, and the corresponding 1D scattering profiles were extracted from the selected zones in the 2D scattering patterns along the in-plane (q_{xy}) and out-of-plane (q_z) directions. The mircobeam (ca. a couple tens of μm) incident angle was set to 0.05° and the sample-to-detector distance was 70.0 mm.

2D GIWAXS patterns were further converted to the scattering vector space, with q_{xy} and q_z representing the scattering vector components respectively along the in-plane and out-of-plane directions; after the conversion, there is a missing wedge of no diffraction information available in the vertical direction of each 2D GIWAXS pattern.[6] According to literature, the missing wedge was caused by the scattering geometry with a fixed-angle incidence.

11. ^{1}H and ^{13}C NMR spectra

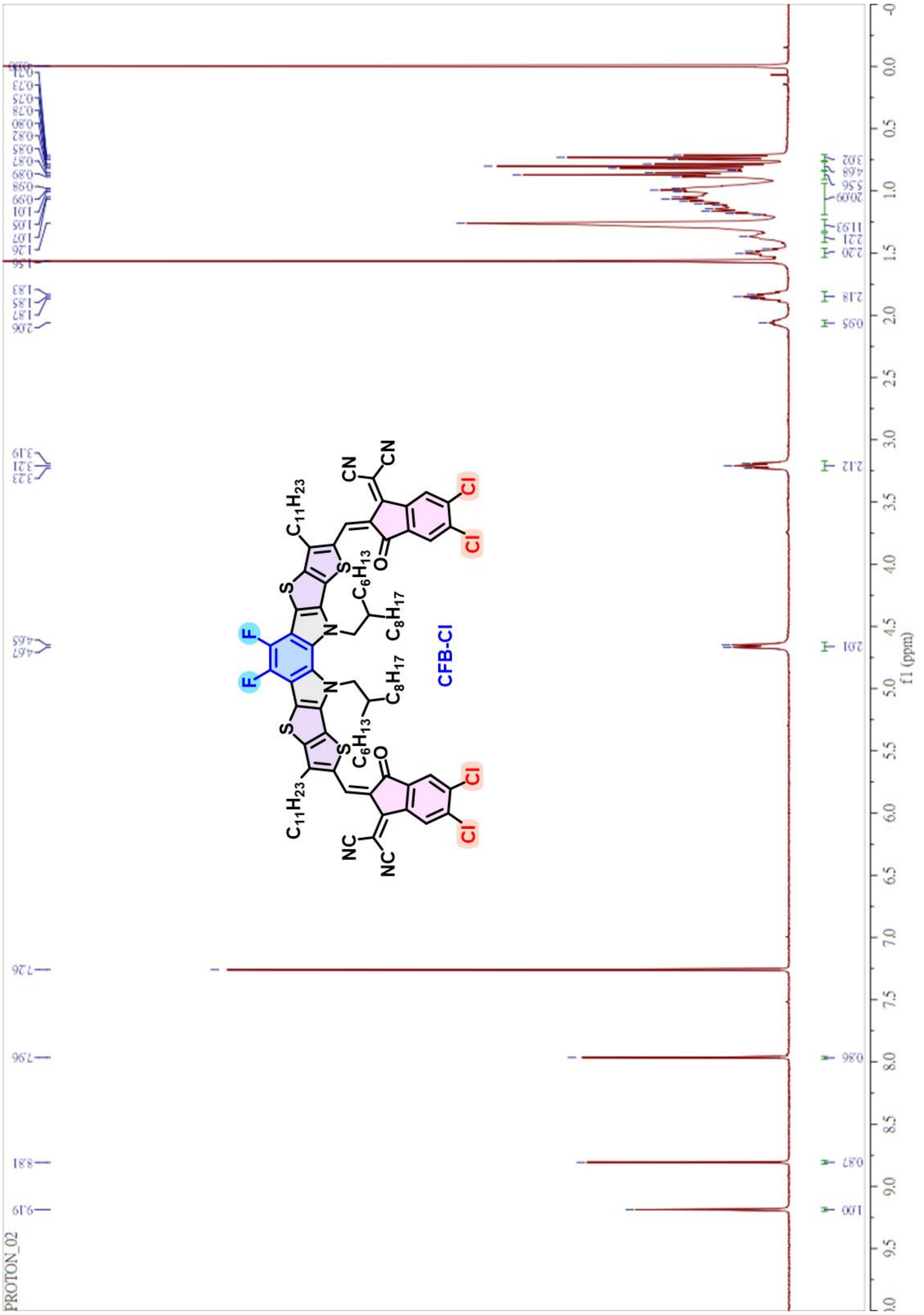

Figure S9. ^{1}H NMR spectra of CFB-Cl.

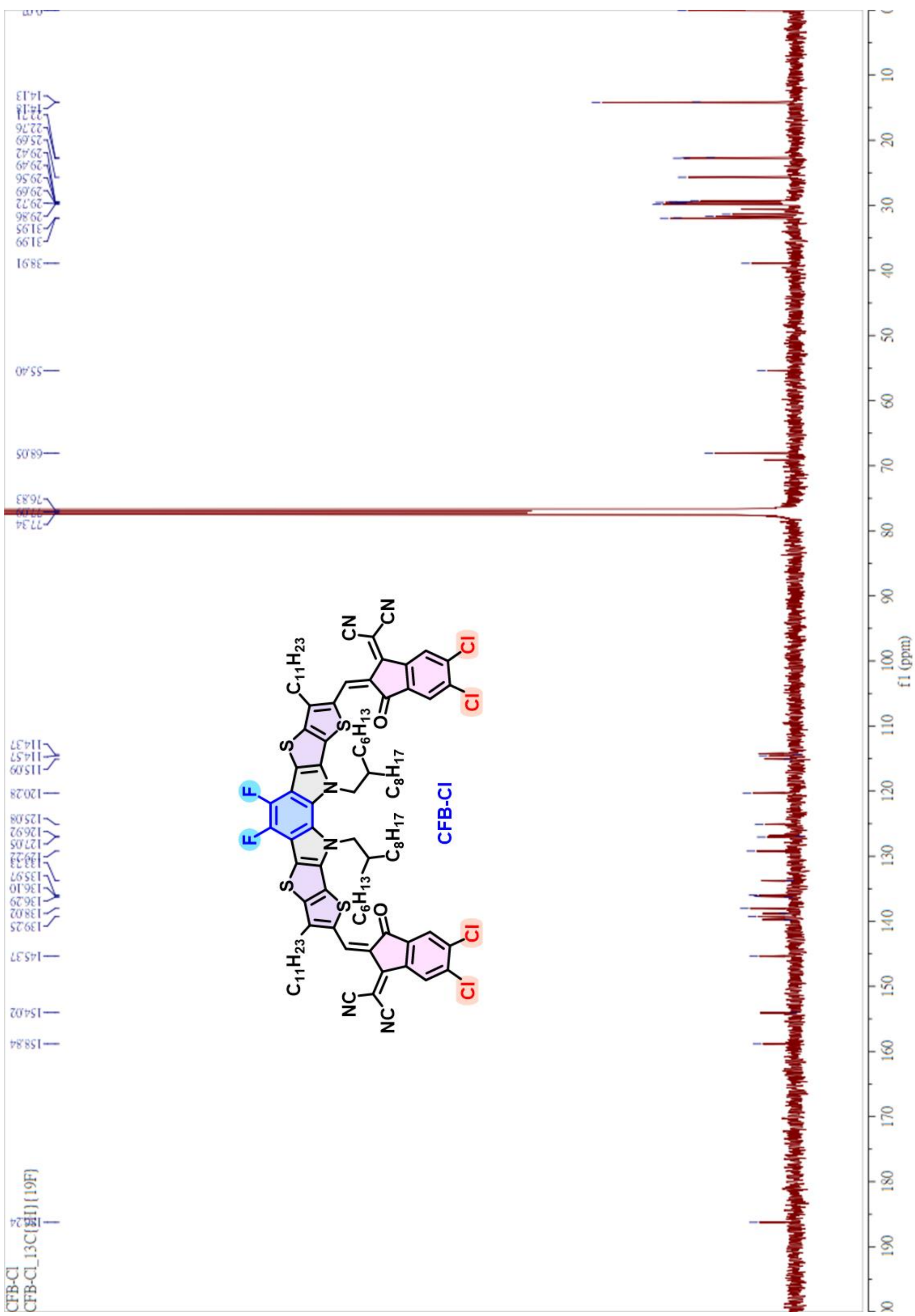

Figure S10. ^{13}C NMR spectra of CFB-Cl.

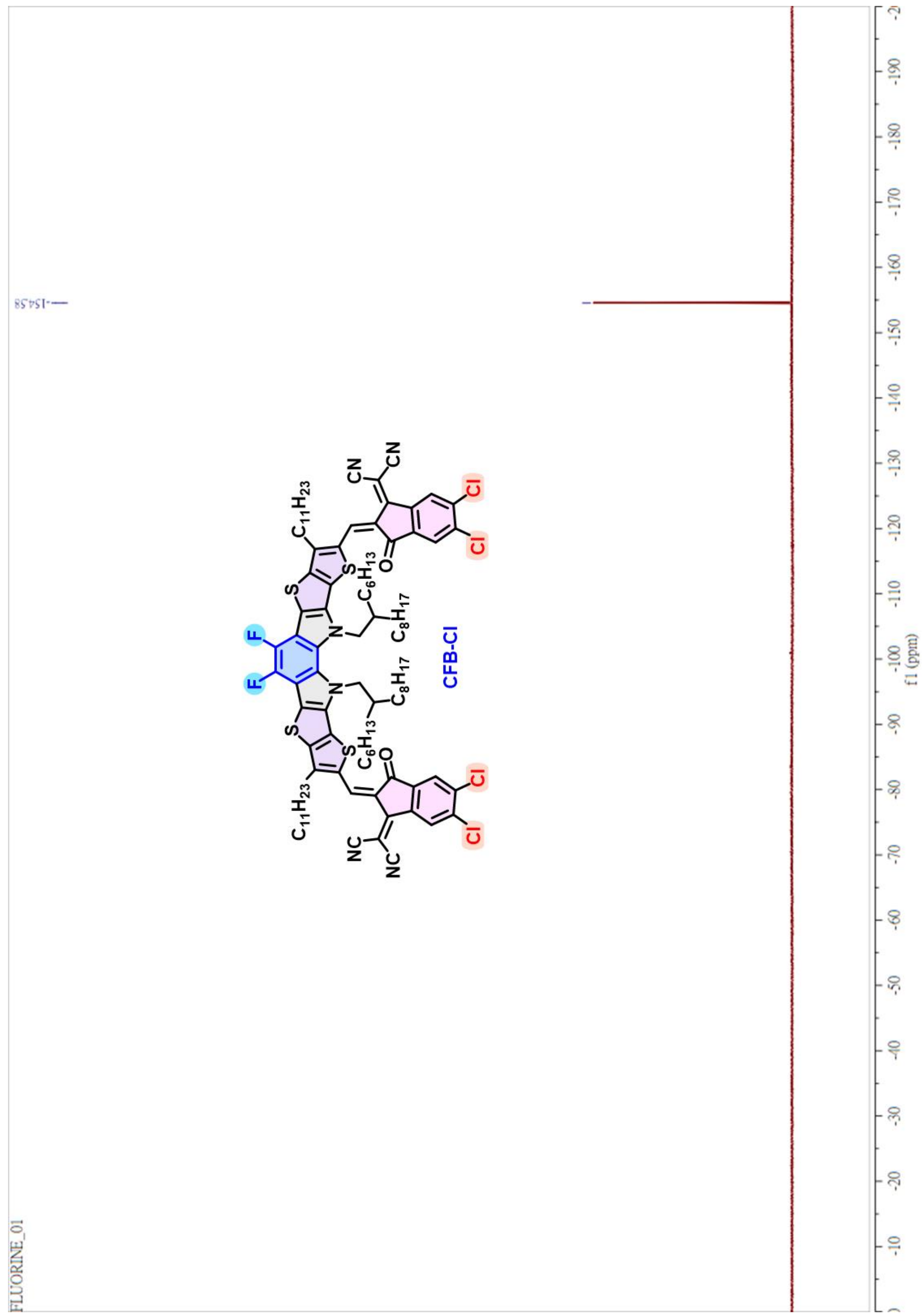

Figure S11. ^{19}F NMR spectra of CFB-Cl.

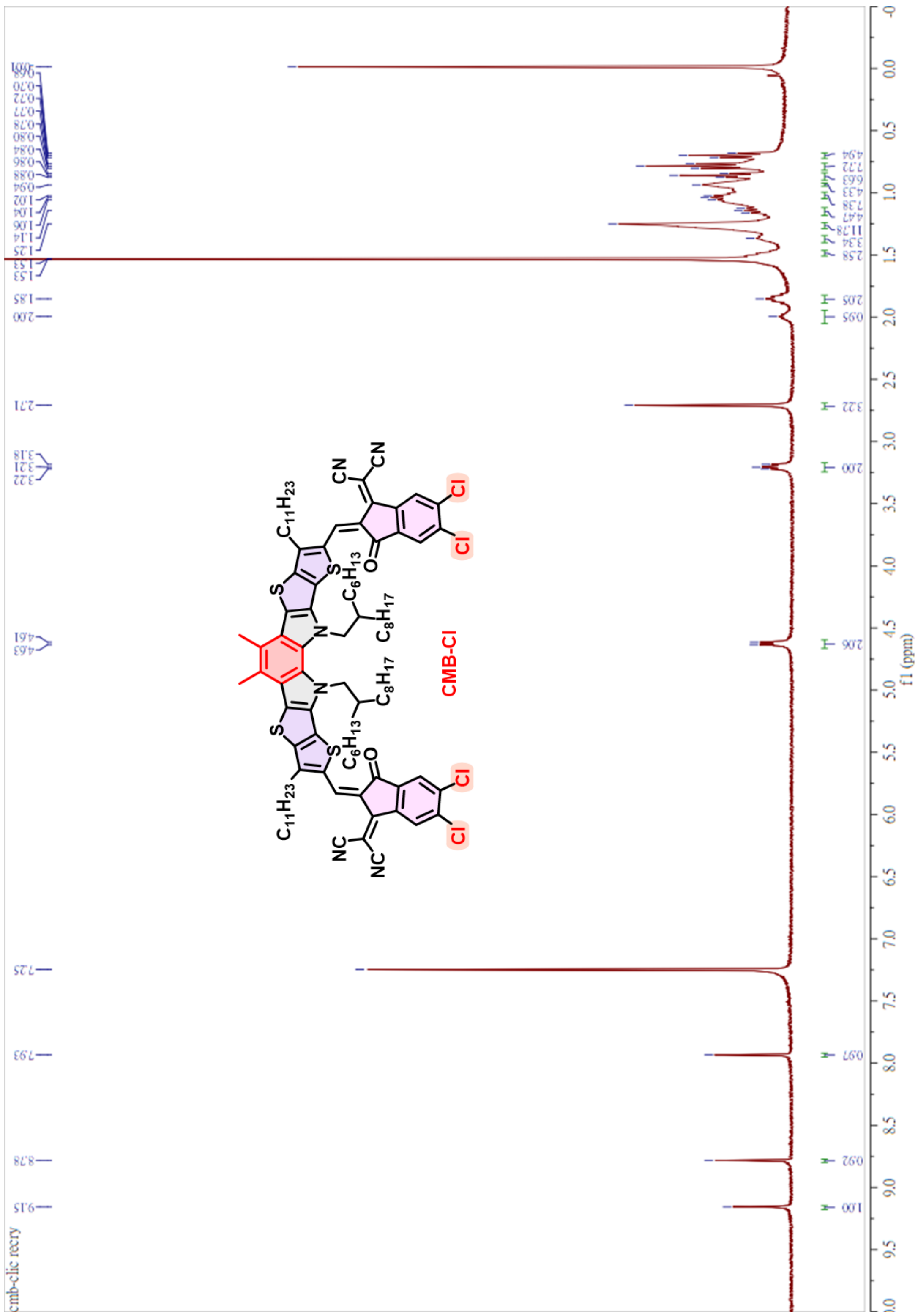

Figure S12. ^{1}H NMR spectra of CMB-Cl.

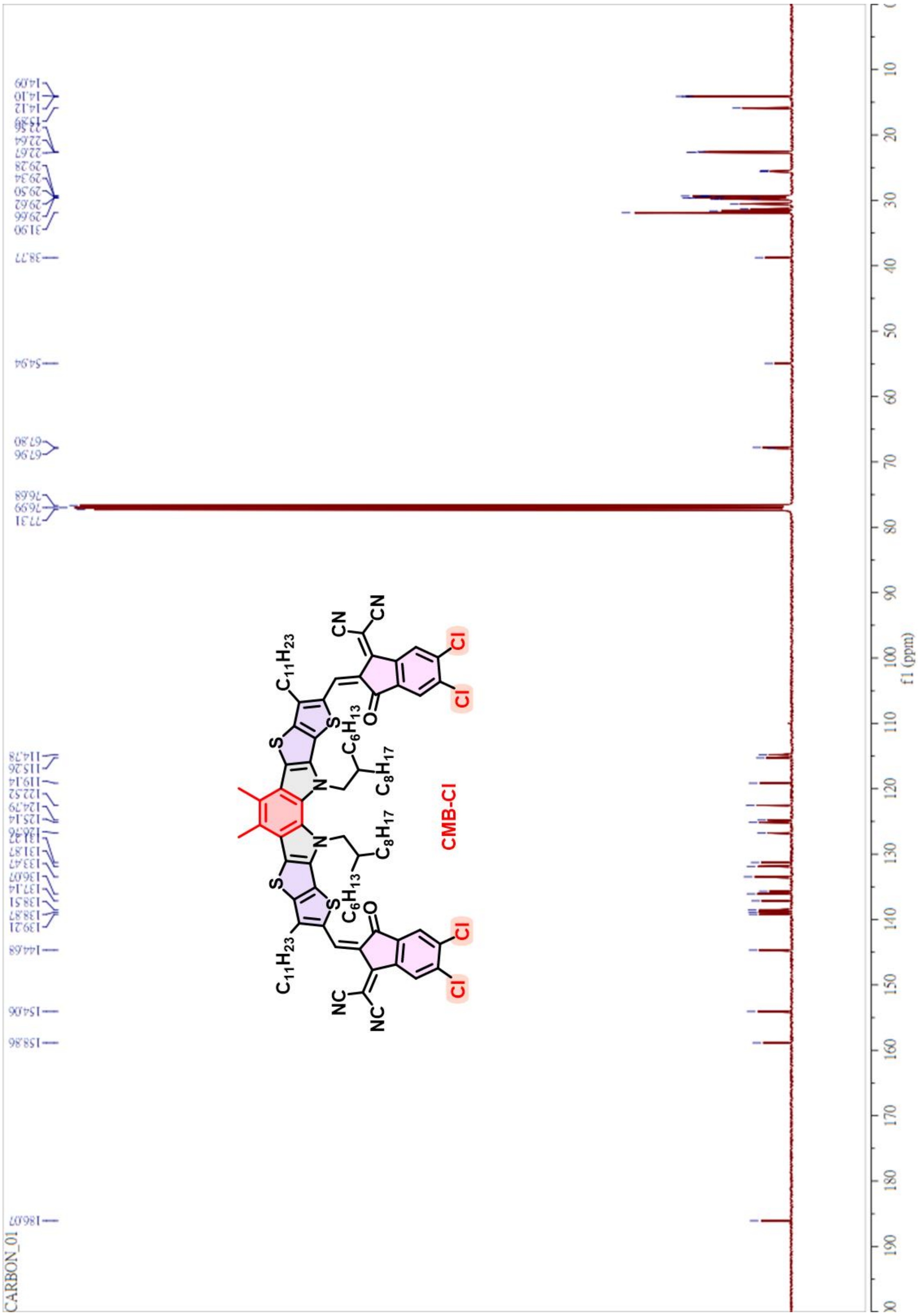

Figure S13. ^{13}C NMR spectra of CMB-Cl.

12. References

[1] L. Krause, R. Herbst-Irmer, G. M. Sheldrick, D. Stalke, Comparison of silver and molybdenum microfocus X-ray sources for single-crystal structure determination. *Appl. Crystallogr.* **2015**, *48*, 3-10.

[2] G. M. Sheldrick, Crystal structure refinement with SHELXL. *Crystal Structure Communications* **2015**, *71*, 3-8.

[3] *APEX4 v.2021.10-0; Bruker AXS GmbH, Karlsruhe, Germany* **2021**.

[4] P. Murgatroyd, Theory of space-charge-limited current enhanced by Frenkel effect. *J. Phys. D: Appl. Phys.* **1970**, *3*, 151.

[5] K.-H. Huang, B.-H. Jiang, H.-C. Lu, Y.-J. Xue, C.-F. Lu, Y.-Y. Chang, C.-L. Huang, S.-Y. Chien, C.-P. Chen, Y.-J. Cheng, Electron-Rich Heptacyclic S,N Heteroacene Enabling C-Shaped A-D-A-type Electron Acceptors With Photoelectric Response beyond 1000 Nm for Highly Sensitive Near-Infrared Photodetectors. *Adv. Sci.* **2025**, *12*, e2413045.

[6] Z. Jiang, GIXSGUI: a MATLAB toolbox for grazing-incidence X-ray scattering data visualization and reduction, and indexing of buried three-dimensional periodic nanostructured films. *Appl. Crystallogr.* **2015**, *48*, 917-926.
